# Native Environment Modulates Leaf Size and Response to Simulated Foliar Shade across Wild Tomato Species

**DOI:** 10.1371/journal.pone.0029570

**Published:** 2012-01-12

**Authors:** Daniel H. Chitwood, Lauren R. Headland, Daniele L. Filiault, Ravi Kumar, José M. Jiménez-Gómez, Amanda V. Schrager, Daniel S. Park, Jie Peng, Neelima R. Sinha, Julin N. Maloof

**Affiliations:** 1 Department of Plant Biology, University of California Davis, Davis, California, United States of America; 2 Department of Plant Sciences, University of California Davis, Davis, California, United States of America; 3 Department of Statistics, University of California Davis, Davis, California, United States of America; University of Chicago, United States of America

## Abstract

The laminae of leaves optimize photosynthetic rates by serving as a platform for both light capture and gas exchange, while minimizing water losses associated with thermoregulation and transpiration. Many have speculated that plants maximize photosynthetic output and minimize associated costs through leaf size, complexity, and shape, but a unifying theory linking the plethora of observed leaf forms with the environment remains elusive. Additionally, the leaf itself is a plastic structure, responsive to its surroundings, further complicating the relationship. Despite extensive knowledge of the genetic mechanisms underlying angiosperm leaf development, little is known about how phenotypic plasticity and selective pressures converge to create the diversity of leaf shapes and sizes across lineages. Here, we use wild tomato accessions, collected from locales with diverse levels of foliar shade, temperature, and precipitation, as a model to assay the extent of shade avoidance in leaf traits and the degree to which these leaf traits correlate with environmental factors. We find that leaf size is correlated with measures of foliar shade across the wild tomato species sampled and that leaf size and serration correlate in a species-dependent fashion with temperature and precipitation. We use far-red induced changes in leaf length as a proxy measure of the shade avoidance response, and find that shade avoidance in leaves negatively correlates with the level of foliar shade recorded at the point of origin of an accession. The direction and magnitude of these correlations varies across the leaf series, suggesting that heterochronic and/or ontogenic programs are a mechanism by which selective pressures can alter leaf size and form. This study highlights the value of wild tomato accessions for studies of both morphological and light-regulated development of compound leaves, and promises to be useful in the future identification of genes regulating potentially adaptive plastic leaf traits.

## Introduction

Plants, as sessile organisms, possess an extraordinary capacity to respond to changing environmental conditions. An example of such phenotypic plasticity is the shade avoidance response, in which plants sense the foliar shade of competitors and respond through internode and leaf elongation, changes in pigment, and reallocation of nutrients, among other traits [1]. Foliar shade is specifically detected through phytochrome receptors, whose activities are modulated by the ratio of red (660–670 nm) to far-red (725–735 nm) wavelengths, but cryptochromes, which measure the intensity of blue light, are important in mediating shade avoidance responses as well [2]–[5]. The ratio of red to far-red wavelengths is indicative of foliar shade as a consequence of red wavelengths being preferentially absorbed by chlorophyll [6]–[7]. As the primary receptive surface for incident light, leaves play an important physiological role in shade avoidance, and at least some phytochrome activity is mediated from the sub-epidermal layers of the blade [8]–[9].

The most stereotypical change in leaves in response to foliar shade is petiole elongation. However, conflicting observations of changes in blade area have been reported in *Arabidopsis* [10]–[12]. When elongation of blade and petiole regions are compared under the condition of darkness followed by monochromatic far-red light, the petiole expands to a greater extent than distal blade regions [3]–[4], [13]. These contrasting responses to far-red light within the leaf are similar to reports of the phenotype of *phyB* mutants, the predominant photoreceptor that mediates shade avoidance in *Arabidopsis* [10]–[12], [14]. However, the exposure to darkness and subsequent monochromatic conditions in these treatments would also activate phyA, the activity of which must be considered as well. Given the difference in how blade and petiole respond to far-red light, it was proposed that selective pressures might independently influence blade and petiole growth [13], [15]–[16]. Some of the genes that might independently regulate the shade avoidance response in petiole regions relative to blade are those known to differentially regulate the growth of the leaf axes. For example, *ANGUSTIFOLIA* regulates growth along the medial-lateral axis of the leaf whereas *ROTUNDIFOLIA3* (*ROT3*) regulates growth along the leaf's proximal-distal axis [17]–[18]. The activity of *ROT3*, a cytochrome family member involved in brassinosteroid biosynthesis, is light-modulated [19]–[20], and other genetic pathways, such as those regulated by gibberellic acid and ethylene, contribute to leaf development through light-dependent and -independent means [2], [21]–[24].

It remains unknown to what degree phenotypic plasticity and adaptation to environmental conditions impinge upon each other in leaves. Plants arising from populations exposed to continuous foliar shade have attenuated shade avoidance responses in their internodes relative to more exposed, pastoral populations [25]–[29]. Similarly, domesticated species often exhibit weak shade avoidance responses, presumably an effect of selection to achieve good yield at higher planting densities [30]. Separate from the shade avoidance response, the question remains: to what extent is leaf size, as an intrinsic developmental trait, modulated by native levels of foliar shade? That is, irrespective of light treatment, is the size of leaves in a population correlated with indigenous vegetation densities? Answering such a question requires both an *in situ* measure of foliar shade and multiple wild populations that can be grown in replicate under controlled conditions. This design ensures that intrinsic developmental traits are not confounded with reaction norms to *in situ* conditions. Since the advent of satellite-derived data, one convenient measure of surface foliar shade levels is the Normalized Difference Vegetation Index (NDVI), which is a ratio of reflected red and far-red wavelengths. The NDVI is proportional to levels of photosynthetically-active canopy, and is therefore a useful indicator of the native foliar shade to which populations may be adapted [31]–[32].

Although high ratios of far-red to red wavelengths are sufficient to experimentally induce the shade avoidance response, in the wild foliar shade is often associated with a number of other environmental changes. Shading by competitors can be detected before changes in light intensity due to reflection of far-red wavelengths from neighbors. However, changes in light quality induced by competitor shading are often associated with decreases in light intensity as well. As might be predicted, morphological differences between shade and sun leaves due to effects of light intensity and altered red to far-red ratios overlap, such that leaves exposed to lower irradiance levels are larger and possess less mass per blade area (LMA, Leaf Mass per Area) [33] to more efficiently intercept filtered light. Indeed, in addition to light quality and quantity, numerous other factors important to the ecophysiology of leaves change with increases in vegetation, including humidity, ambient air temperature, wind speed, precipitation, and soil moisture content [34]–[35]. This constellation of environmental variables that are affected by vegetation density are of concern to those who model leaf size as an adaptive feature that maximizes photosynthetic output while minimizing water loss through transpiration [36]–[38]. Although model results vary, the general consensus from empirical observations is that in order to maximize water use efficiency, both temperature and light intensity must be considered, and that larger leaves tend to be favored in warmer environments with low light intensity [39]–[44].

So robust is the relationship between the environment and leaf form that paleobotanical and paleoclimactic studies routinely use leaf area and the number and depth of serrations in fossil leaves as measures of ancient climatic conditions. Larger, entire (smoothened margin) leaves are known to be more prevalent in warmer, wetter environments with the converse being true for drier climates [45]–[50]. Confirmation of this correlation has been tested in extant populations [51]–[54], and evidence for both intrinsic genetic determination and phenotypic plasticity is apparent [55]. This distinction between intrinsic developmental variation and phenotypic plasticity is important when analyzing the changes in fossil leaf shape over evolutionary time. Whereas plastic responses to the environment occur throughout the lifetime of the organism, static adaptations occur over generational timescales through natural selection, and may lag in their response to rapid climate change [55].

Here, we analyze wild tomato accessions (*Solanum* Sect. *Lycopersicon*) derived from native populations in Peru and Ecuador [56] that inhabit locales with diverse levels of foliar shade as measured by NDVI and that vary in their levels of precipitation and temperature. We measure a variety of leaf traits from these accessions under controlled light conditions with different red-to-far red ratios and find that leaf dimensions and area correlate with native environmental conditions. Additionally, the plastic response of accessions to far-red light treatments correlates with native levels of foliar shade. We characterize the nature of the shade avoidance response across the leaf series and along the leaf length, and find that the degree and direction of the correlation of leaf traits with NDVI values may be modulated through heterochronic or ontogenic means. Our results provide an example of how leaf size and morphology is influenced by environment at both inter- and intra-species levels and demonstrate the importance local adaptation and plasticity play in shaping leaf forms.

## Materials and Methods

### *Solanum* Sect. *Lycopersicon* accessions used in this study

In this study, we look at the relationship between the value of environmental variables present at the point of collection of wild tomato accessions and a number of leaf traits measured under simulated foliar shade or sun conditions. The environmental variables used in this study are derived from Nakazato et al. [57], and include Normalized Difference Vegetation Index (NDVI, a satellite measure of the amount of vegetation present in a locale), longitudinal and latitudinal coordinates, altitude, mean annual temperature, and mean annual precipitation. Accessions were obtained from the C.M. Rick Tomato Genetics Resource Center (TGRC, U.C. Davis), which maintains accessions as an out-crossing population of ten individuals. The extensive germplasm of the TGRC and the corresponding documentation of the accessions held there [57] provide an excellent opportunity to study relationships between the environment and plant development. It is possible that the values of environmental variables at the point-of-origin of accessions have changed since their sampling, leading to discrepancies between the actual environment accessions are derived from and the environmental values reported in this study. However the strong environmental correlations that we find suggest that on average such changes are relatively small and this is a valid resource for studying environment/development relationships.

Wild tomato accessions were selected for this study using a D-optimal design, in which simulated annealing was used to maximize the determinant of the Fisher information matrix. An optimal design approach was used to maximize the spread of longitude, latitude, altitude, and NDVI values present in the accessions studied. This was done to estimate correlation between accession traits and environmental variables more efficiently, requiring fewer sampled accessions to precisely measure correlation than if an optimal design had not been used. Importantly, because of the simulated annealing, the outcome of D-optimal design changes each time it is performed. Thus, the accessions chosen from the D-optimal design are random within the constraints of the procedure, impacting our decision to use accession as a random variable in mixed-effect linear models (see *Statistical modeling*, below).

### Growth conditions

Plants were grown in a (Conviron) walk-in chamber in which the left and right sides were divided into low and high red-to-far red light treatments, which we refer to as “simulated foliar shade” and “simulated sun.” Each side consisted of six shelves fitting five 11″×22″ inch trays each. Temperature was adjusted to 22°C and photoperiod to a 16∶8 hour light-dark cycle. Lighting consisted of alternating fluorescent (F48T12CWHO) and far-red (F48T12FRHO, peak emission 750 nm, Interlectric, Warren, PA) bulbs. High red-to-far red wavelength ratios were achieved by blocking far-red irradiance with sleeves whereas all bulbs (both normal fluorescent and far-red) transmitted light in the low red-to-far red treatment. Shade cover was placed perpendicularly over bulbs in the low red-to-far red treatment (simulated foliar shade) to adjust overall PAR (Photosynthetically Active Radiation) to match that of the high red-to-far red treatment (simulated sun).

Wild tomato seed was sterilized for 10 minutes in 50% bleach, washed in autoclaved water, and subsequently plated on 1/2MS plates. Plates were placed in darkness covered in foil at ambient room temperature for three days and then placed into the simulated sun treatment for nine days, after which seedlings were transferred one per ∼5″×5″ inch pots placed eight per 11″×22″ inch trays in Sunshine (SunGro) soil mix and moved to experimental light conditions. Plants were watered by filling trays with just enough water to cover the bottoms of pots and waiting for water to evaporate before watering again. Plants were harvested for phenotypic analysis starting 28 days post-plating.

Two experiments were performed to replicate results, and the sides of the chamber used for simulated sun and simulated foliar shade treatments were switched between each experiment. Up to 10 seedlings per accession per treatment per experiment were transplanted for analysis. The mean and median numbers of plants analyzed under the simulated sun treatment overall for each accession was 15.3 and 16, respectively; for the simulated foliar shade treatment these statistics were 14.9 and 15, respectively. 726 plants were analyzed in total.

### Photography

At the time of harvest, only the first four leaves of each plant had expanded sufficiently for analysis, and the adaxial and abaxial sides of leaves were photographed. In addition the primary and terminal leaflets were removed and photographed alone. Olympus SP-500 UZ cameras were mounted on copy stands (Adorama, 36″ Deluxe Copy Stand) and controlled remotely by computer using Cam2Com software (Sabsik).

### Trait measurement

Leaf Number was counted at the time of harvest and included young primordia that could be observed by eye (∼2–3 mm in overall length) that likely corresponded to P4–P5 (that is, the fourth and fifth oldest leaf primordia).

Lengths of leaf sub-regions were determined using ImageJ [58] and converted to absolute length values as measured by a ruler present in every photograph. Sum Length, Sum Width, and individual lengths of leaves were measured using LAMINA [59]. The correlation coefficient between manually measured lengths in ImageJ and semi-automatic length measurement by LAMINA was >0.99. Leaf/leaflet area measurements were made using standard ImageJ functions.

The Red-to-Green Ratio of leaves, as determined from photographs taken under controlled lighting conditions, was measured from the abaxial side of leaf series. Average R, G, and B values of pixels (RGB, a digital color model) of individual leaves were recorded by selecting leaves using an appropriate tolerance value to separate them from the white background in Photoshop (Adobe). The Red-to-Green Ratio represents the sum of R values across the first four leaves divided by the sum of G values of the first four leaves. In addition to controlled lighting conditions and camera parameters, the accuracy of this value is further buffered by the fact it is a ratio largely independent of light intensity values.

### Statistical modeling

Mixed effect linear modeling was used to obtain fitted values of accessions under each light treatment to not only account for noise in the data, but most importantly to provide developmental rate corrections. Different accessions of wild tomato species have varying developmental rates, which we measured by Leaf Number (**Fig. S1**). As an alternative we could have developmentally staged accessions to ensure measurements between accessions were comparable, as Leaf Number is highly correlated with a number of the traits we measured (**Fig. S2**). We opted not to do this, because 1) there are many different, and often incongruent, criteria by which plants could be developmentally staged, and 2) staging is an error-prone, subjective endeavor.

Mixed-effect linear models were fit using the lme4 package in R [60]–[61]. For traits in which the unit measure is squared, such as Sum Area, Leaflet Area, and Perimeter^2^/Area, the square root was taken before modeling to provide a more normal distribution. Models were selected through a process of backwards selection, in which two models differing by only the presence of a single term were compared to determine the significance of the term in explaining variance in the data. The process was repeated for all terms (replacing the previously tested term and testing another) and at the end of the process the most non-significant term (using a p-value threshold of 0.05) was removed from the model. This process was iterated until only significant terms remained in the model. We then performed a forward selection check of the resulting minimal model, adding terms previously removed back to the model and comparing to the minimal model to ensure that the non-significance of removed terms persists.

Maximal models included the following fixed effects: species and light treatment. The random effects used in maximal models were the following: accession, natural log (ln) of Leaf Number, tray, shelf, time of harvest, camera used to photograph, and experiment (replicate). Of special note, we use accession as a random effect despite accessions within a species being chosen by a D-optimal design, which chooses accessions such that they maximize the determinant of the Fisher information matrix. Because a simulated annealing procedure is used in this method, the accessions chosen each time by the procedure will change. Therefore, accessions are randomly chosen, within the constraints of the D-optimal design procedure used, and results from this study should be interpreted within this context. The ln of Leaf Number was used to provide linearity with respect to various leaf dimension traits (see **Fig. S2**). Shelf, time of harvest, camera used to photograph, and experiment were never significant terms of models, with the exception of experiment with respect to the Red-to-Green Ratio of leaves. Light treatment and accession were significant terms for all traits analyzed in the study. For leaf dimension traits (Sum Length, Sum Width, (Sum Area)^1/2^, and (Leaflet Area)^1/2^), significant terms in the models included the following: light treatment, species, accession, ln(Leaf Number), and tray (although tray was not significant with respect to (Leaflet Area)^1/2^). For models of the lengths, sub-lengths, and areas of individual leaves, these same terms were significant for each leaf and sub-leaf region, and were included in each model across the leaf series. Significant terms included in models of Leaf and Leaflet Perimeter/(Area)^1/2^ and the Red-to-Green Ratio of leaves varied. A complete list of terms used in final minimal models for each trait and their significance is given in **Table S1**.

Fitted values for accessions under the simulated sun light treatment were derived from modeled intercept values. The simulated foliar shade treatment values were calculated as the simulated sun value for each accession plus the effect of the simulated foliar shade treatment. The “shade avoidance response” for each trait was then calculated as the ratio of the simulated foliar shade trait value divided by the simulated sun trait value for each accession. If X_SUN_ is the modeled intercept value for a trait in simulated sun, X_SHA_ the resulting calculated simulated shade trait value, and “l” the effect of light, then the shade avoidance response = X_SHA_/X_SUN_ = (X_SUN_+l)/X_SUN_. Correlation between environmental variables and trait values under each light treatment or the shade avoidance response was then analyzed.

Because our models are developmental rate corrected, the effect of simulated foliar shade treatment is independent of leaf size; that is, changes in leaf dimensions in response to simulated shade treatment are fixed, rather than proportional to the size of a leaf. The validity of this assumption is reflected in the raw data (see **Fig. S3**), in which a regression model of accession leaf length in simulated shade versus its length in simulated sun has a slope that is statistically indistinguishable from 1 (p = 0.23). If the slope had a value significantly larger than 1, then responses in leaf length to simulated shade would be proportional to the size of leaves under the simulated sun treatment, but this is not the case. The fixed increases in leaf dimensions in response to simulated foliar shade, regardless of the developmentally intrinsic size of leaves, creates a situation in which larger leaves have a proportionally smaller response to simulated foliar shade relative to smaller leaves. We discuss at length in the Results and Discussion section how the aforementioned relationships create predictable outcomes between the correlation of leaf dimensions versus shade avoidance response with environmental variables.

## Results and Discussion

### Environmental context of accessions

**Figure 1:**
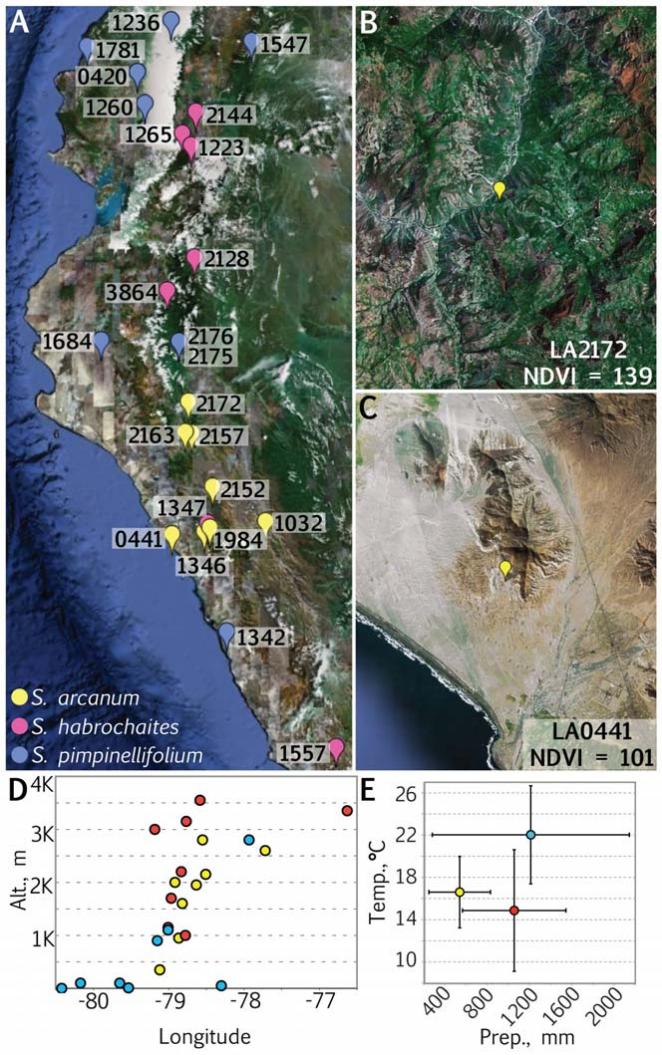
Native environments of wild tomato accessions. A) Accessions of *S. arcanum* (yellow), *S. habrochaites* (red), and *S. pimpinellifolium* (blue) originate from locales ranging from the coastline to the Andes mountain range in Ecuador and Peru. Numbers correspond to accession numbers. NDVI (Normalized Difference Vegetation Index) values are satellite-derived measures of surface foliar shade. Of the *S. arcanum* accessions used in this study, LA2172 (B) has the highest the NDVI value and LA0441 (C) the lowest. Note the increased foliage surrounding the location of LA2172 relative to LA0441. D) *S. pimpinellifolium* accessions uniquely occupy lower elevation levels near the coastline relative to accessions from other species analyzed. E) Averaged annual mean temperature and precipitation levels of accessions from each species demonstrate the unique environments from which accessions arise. Bars represent SD.

To determine whether leaf traits and shade avoidance response in leaves correlate with native levels of foliar shade, we analyzed wild tomato accessions obtained from the extensive germplasm resources of the Tomato Genetic Resource Center (TGRC, U.C. Davis). For three wild tomato species represented in the TGRC, accessions were chosen using a D-optimal design such that a better spread of longitude, latitude, altitude, and NDVI values were represented among accessions. This allowed more efficient estimation of correlations between traits and environmental parameters using fewer sampled accessions (**Fig. 1A**, **Table S2**).

NDVI values are directly proportional to the photosynthetic capacity of canopies, and can therefore serve as useful indicators of the foliar shade that accessions experience *in situ* [31]–[32]. Higher NDVI values represent land surfaces with low reflectance of red wavelengths relative to far-red, due to absorption of red wavelengths by vegetation. For the sampled accessions, NDVI values qualitatively correlate with images of vegetation in the visible spectrum from satellite imagery (**Fig. 1B, C**). Besides NDVI, we analyzed the correlations of leaf traits with other environmental variables, including longitude, latitude, altitude, mean annual precipitation, and mean annual temperature [57] (**Table S2A**). Some of these variables had expected significant correlations with each other (for example, altitude and temperature are significantly negatively correlated, and altitude and longitude are positively correlated because of the Andes mountain range; **Table S2B**). Although the average NDVI values were not significantly different between species, most other variables showed species-specific biases (**Fig. 1D, E**) [56]. Most notably, the *S. pimpinellifolium* accessions studied occupy coastal regions of a lower altitude compared to other species (the only five accessions studied with altitudes <100 m and four accessions with longitudes <−79.3 are all *S. pimpinellifolium*; **Fig. 1D**).

### Correlation of leaf traits with NDVI

Although numerous studies have demonstrated that populations occupying highly vegetated areas have reduced shade avoidance responses relative to populations in exposed areas [25]–[29], how developmentally intrinsic leaf traits correlate with levels of foliar shade remains understudied. The final dimensions of a leaf presumably reflect the intersecting influences of plastic response to environmental conditions and other developmental pathways insensitive to environmental inputs.

**Figure 2:**
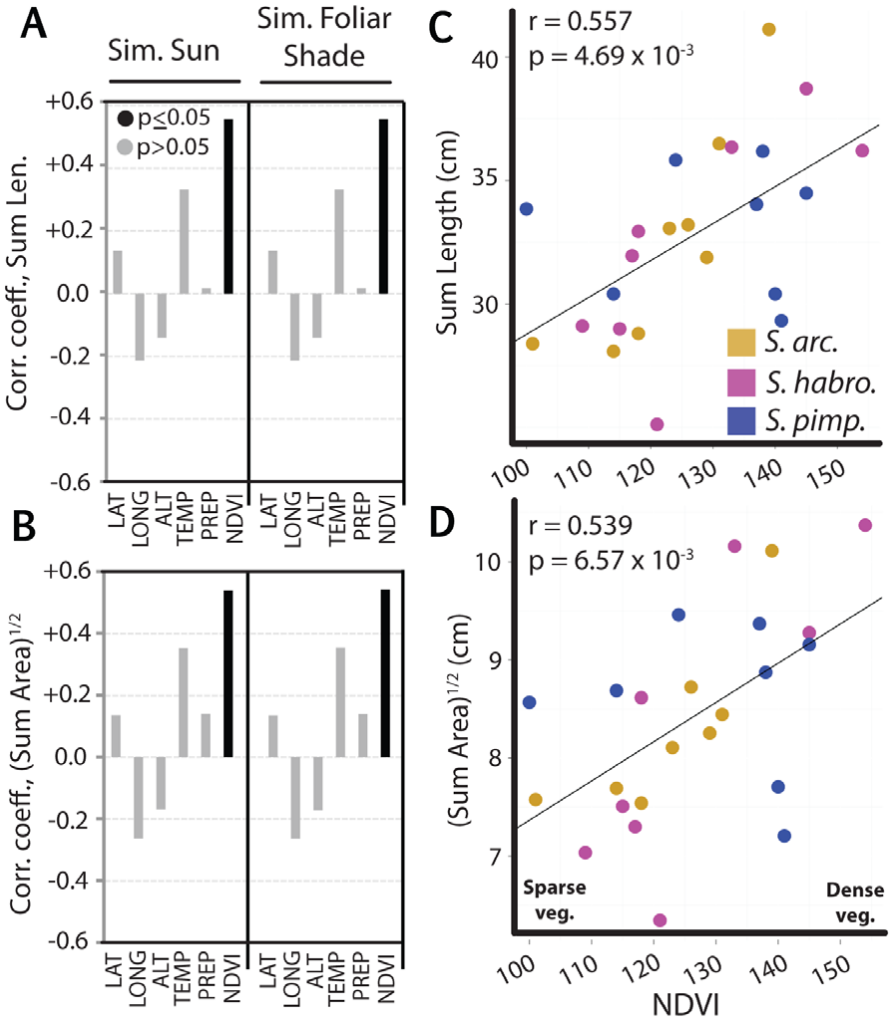
Correlation of traits with environmental variables. *r* values (Pearson) representing correlations between A) Sum Length and B) (Sum Area)^1/2^ of accessions with latitude, longitude, altitude, mean annual temperature, mean annual precipitation, and NDVI. Correlation coefficients are given for accessions of all species under simulated shade and sun conditions. Scatter plots showing the positive correlations between C) Sum Length and D) (Sum Area)^1/2^ with NDVI under simulated sun conditions. Significance of *r* values deviating from 0 is denoted by solid (p<0.05) and gray (p>0.05) fill. *S. arcanum*, *S. habrochaites*, and *S. pimpinellifolium* accessions are represented by gold, magenta, and navy, respectively.

**Figure 3:**
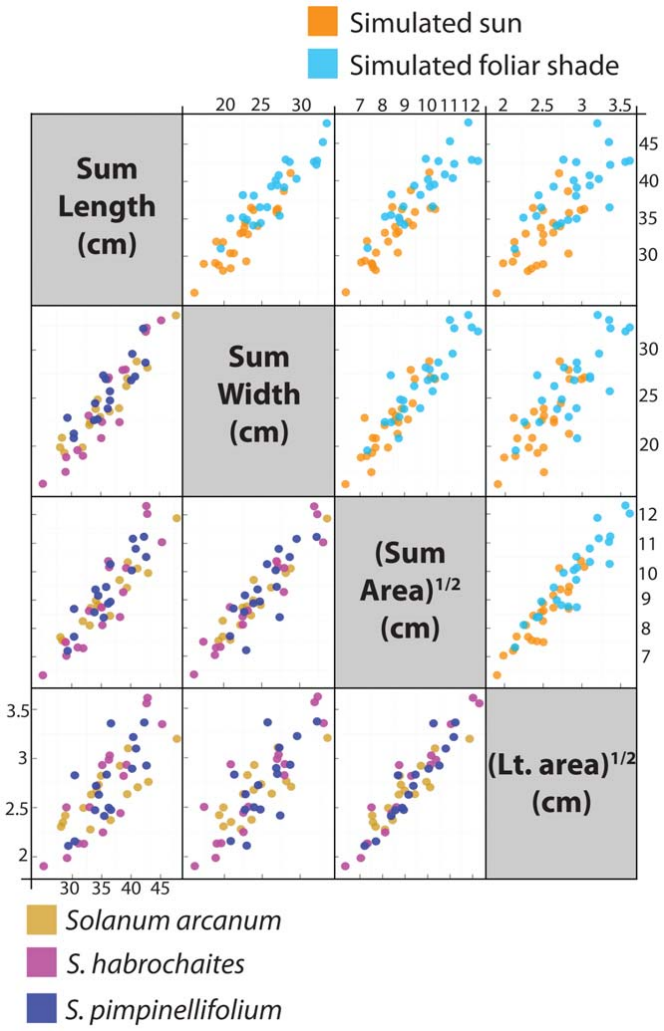
Correlations between leaf dimension traits by light treatment and species. Pair-wise scatter plots between leaf dimension traits, including Sum Length, Sum Width, (Sum Area)^1/2^, and (Lft. Area)^1/2^. Each accession is represented by two points in each plot: once for simulated shade values and once for simulated sun values. In the upper right panels, points are colored by light treatment (orange, simulated sun; cyan, simulated shade). In the lower left panels, points are colored by species (gold, *S. arcanum*; magenta, *S. habrochaites*; navy, *S. pimpinellifolium*).

Irrespective of light treatment, the sum of leaf traits across the first four leaves, Sum Length and (Sum Area)^1/2^ (**Fig. S4**), are significantly positively correlated with NDVI (**Fig. 2**). Another way to view the relationships between environmental variables and leaf traits such as dimensions and area (which are themselves highly correlated, **Fig. 3**) is using Principal Component Analysis (PCA). PCA performed on Sum Length, Sum Width, (Sum Area)^1/2^ and (Leaflet Area)^1/2^, with each accession represented twice (once for its simulated sun and once for its simulated foliar shade trait values) yields a first principal component (PC1) explaining 91% of all variance (**Fig. S5**). PC1 largely represents leaf size, and its factor loadings for (Sum Area)^1/2^, Sum Length, Sum Width , and (Leaflet Area)^1/2^ are 0.52, 0.49, 0.50, and 0.49, respectively. Contrastingly, PC2 mainly represents the length and area of leaflets, and the respective PC2 loadings are −0.19, 0.55, 0.35, and −0.72. Simulated sun or simulated foliar shade treatment PC1 values for accessions are most highly correlated with NDVI relative to other environmental factors, demonstrating the strong relationship between leaf dimensions and vegetation density.

Leaf Number, a measure of developmental rate, has a nearly significant correlation with NDVI in each treatment (p<0.07) and is significantly correlated with NDVI if only *S. arcanum* and *S. habrochaites* accessions are considered (**Table 1**). This correlation highlights the importance of correcting for developmental timing effects. Leaf Number is negatively correlated with NDVI (**Table 1**) whereas leaf dimension positively correlates with it (**Fig. 2**), despite the positive correlation between leaf size and number (**Fig. S2**). Although we can only hypothesize if these correlations with NDVI represent adaptation to levels of foliar shade or other associated factors, it is tempting to speculate that increases in leaf area and longer plastochron intervals (that is, slower developmental rate) might confer a fitness advantage to accessions in regions with high foliage by providing increased blade area for light capture and/or allowing for the proper positioning of laminae to capture light filtering through canopy.

**Table 1:** Correlation between leaf number and environmental variables.

	All Species				*S. arc.* and *S. habro.* Only			
	Lat.	Long.	Alt.	NDVI	Lat.	Long.	Alt.	NDVI
**Sim. Sun**	0.256	−0.298	−0.260	−0.385	0.093	−0.135	0.030	−0.636**
**Sim. Shade**	0.256	−0.298	−0.260	−0.385	0.093	−0.135	0.030	−0.636**
**Shade/Sun**	0.259	−0.282	−0.235	−0.357	0.112	−0.123	0.065	−0.587*

*r* values (Pearson) representing correlation between Leaf Number (LFN) values with environmental variables. Correlation coefficients are given for simulated shade and sun conditions and the ratio of LFN between these treatments (Shade/Sun). Correlation is between either accessions of all species or only accessions of *S. arcanum* and *S. habrochaites*. Note the negative correlation between developmental rate and NDVI values under each light treatment and in response to simulated foliar shade. Correlation becomes significant when only *S. arcanum* and *S. habrochaites* accessions are considered. *p<0.05, **p<0.01, ***p<0.001.

### Correlation of leaf traits with temperature and precipitation

Although our measurements of leaf traits were designed with the ultimate goal of determining norm of reactions to differing far-red light treatments, we were nonetheless interested in whether traits correlated with other biologically-relevant environmental variables, such as precipitation and temperature. Together with light intensity, precipitation and temperature bear heavily on current theory concerning adaptive leaf sizing with regard to water use efficiency. For example, modeling of optimal photosynthetic rates in leaves suggests that water use efficiency increases for larger leaves when absorbed radiation is low; conversely, water use efficiency is greatest for smaller leaves when absorbed radiation is high [36]–[37]. Furthermore, temperature restricts the range of leaf sizes under which water use efficiency can be significantly modulated, such that water use efficiency is maximized for smaller leaves in colder environments, regardless of light intensity.

The strongest empirical evidence for the adaptive significance of leaf size and shape comes from the fossil record. Paleobotanical and paleoclimatic studies often rely on a positive correlation between warmer, wetter climates and larger leaves with smoother margins to deduce ancient meteorological conditions [45]–[50]. These correlations have been verified at an inter- and intra-species level in present day, wild populations [51]–[54]. However, studies of both the fossil record and wild populations confound developmentally intrinsic leaf traits with norms of reaction, and are subject to the influence of numerous unknown environmental factors [55].

Our data supports a positive correlation between leaf size and temperature in wild tomato, similar to the conventions used to deduce ancient climates from fossil foliage. The correlation coefficients between Sum Length, Sum Width, and (Sum Area)^1/2^ with mean annual temperature for accessions of all species are the highest observed after that with NDVI, and are nearly significant (**Fig. 2**, **Table 2**). These correlations, however, do become significant when *S. arcanum* and *S. habrochaites* are considered alone. Correlations between leaf dimensions and mean annual precipitation meanwhile did not reach the same levels of significance.

**Table 2:** Correlation of traits with mean annual precipitation and temperature.

	All Species				*S. arc.* and *S. habro.* Only			
	Prep.		Temp.		Prep.		Temp.	
	Sun	Shade	Sun	Shade	Sun	Shade	Sun	Shade
**Sum Length**	0.017	0.017	0.332	0.332	0.368	0.368	0.603*	0.693**
**Sum Width**	0.157	0.160	0.412*	0.406*	0.366	0.370	0.651**	0.633**
**LFN**	−0.015	−0.015	0.105	0.105	−0.373	−0.373	−0.274	−0.274
**(Sum Area)^1/2^**	0.141	0.140	0.353	0.355	0.445	0.446	0.612*	0.608*
**(Lft. Area)^1/2^**	0.361	0.367	0.356	0.382	0.388	0.421	0.697**	0.625**

Correlation coefficients between leaf dimension traits and mean annual precipitation and temperature. After NDVI, temperature is the most correlative variable with leaf dimension traits, becoming significant when only *S. arcanum* and *S. habrochaites* accessions are considered. *p<0.05, **p<0.01, ***p<0.001.

To measure morphological traits of leaves, we analyzed the value perimeter/(area)^1/2^ (PSQA) for both leaves and leaflets. For whole leaves, this value increases the more complex and serrated or lobed the leaf is, while for leaflets the value increases with the amount of lobing or serration only. Importantly, serration number and depth are features that correlate with temperature and precipitation over geologic time. Although leaf and leaflet PSQA values are significantly changed in response to simulated foliar shade, the size of the effect is less compared to other traits examined (**Fig. S6**). Leaf PSQA values of all accessions have significant correlation with mean annual precipitation but those for *S. pimpinellifolium* are particularly strong (**Table 3**). Additionally, considering the PSQA values of leaflets, *S. pimpinellifolium* was the only species to show a strong significant correlation with temperature, while *S. arcanum* and *S. habrochaites* showed no significant correlation.

**Table 3:** Correlation of leaf and leaflet PSQA with mean annual precipitation and temperature.

	All Species				*S. pimpinellifolium* Only			
	Prep.		Temp.		Prep.		Temp.	
	Sun	Shade	Sun	Shade	Sun	Shade	Sun	Shade
**Leaf**	−0.437*	−0.437*	−0.166	−0.166	−0.751*	−0.751*	−0.539	−0.539
**Lft.**	−0.157	−0.153	0.009	0.006	−0.575	−0.574	−0.715*	−0.722*

Reflecting trends from the paleobotanical record, perimeter/(area)^1/2^ (PSQA) values correlate with mean annual temperature and precipitation levels from the point-of-origin of accessions. Leaf PSQA values correlate to a higher degree with precipitation than leaflet PSQA values, which correlate more highly with temperature in *S. pimpinellifolium* accessions. The data may suggest that leaf complexity and leaflet serration are indicators of precipitation and temperature, respectively, and species-specific adaptation with respect to *S. pimpinellifolium*. *p<0.05, **p<0.01, ***p<0.001.

Overall, the correlations between leaf size, developmental rate, and measures of leaf morphology with environmental variables all suggest adaptation specific to different groupings of species. In all the significant species-specific correlations we observe, *S. arcanum* and *S. habrochaites* form a distinct group from *S. pimpinellifolium* accessions. As mentioned previously, *S. pimpinellifolium* occupies a unique environmental niche characterized by low altitudes (**Fig. 1D**) and relatively higher precipitation and temperature (**Fig. 1E**) in the northern coastal region of Peru (**Fig. 1A**). Of the 24 accessions sampled, five of the eight *S. pimpinellifolium* accessions occupy the lowest altitudes represented. Morphologically, the leaves of *S. pimpinellifolium* accessions possess a defining attribute relative to other wild tomato species: their leaflets are distinctly cordate, or heart-shaped [62].

Given the distinct niche occupied by *S. pimpinellifolium*, the cordate morphology of its leaflets may represent a proxy increase in blade area via morphology. Givnish and Vermeij [37] give special consideration to the cordate leaves they observe in understory vines, interpreting the cordate form as the opposite of dissection, as a means to obtain increased blade area per laminar unit. Additionally, they view cordate leaves as a means to support leaves on erect petioles, which could feasibly function to precisely place leaves to capture understory filtered light. Within this leaflet morph, *S. pimpinellifolium* accessions might adapt to their environments by changes in leaflet serration, accounting for the *S. pimpinellifolium*-specific correlations between leaflet PSQA and temperature. *S. arcanum* and *S. habrochaites* accessions, contrastingly, possess non-cordate leaves and might alternatively modulate the water use efficiency of their leaves through actual changes in leaf size [36]–[37]. The constraints that the relatively lower temperatures of their habitats place on the size of their leaves might already be fulfilled by the absence of the cordate morph. Alternatively, smaller leaf size in these accessions might be attainted through slower developmental rates, as indicated by the unique correlation in these accessions between Leaf Number and NDVI (**Table 1**).

Although exploratory, our data demonstrate phenotypic divisions within the tomato clade potentially indicative of different adaptation strategies that depend on the interplay between leaf size, morphology, and the environment.

### Plasticity of leaf traits under simulated sun and foliar shade conditions

To understand how the shade avoidance response manifests itself in tomato leaves, leaf dimensions were measured under simulated sun and foliar shade conditions. Both the linear dimensions (Sum Length and Sum Width) of leaves and the area ((Sum Area)^1/2^ and (Leaflet Area)^1/2^ of the most distal primary leaflets of leaf 3, see **Fig. S4**) significantly increased in simulated foliar shade conditions relative to simulated sun (**Fig. 3**). Because Leaf Number of each plant was included as a factor in the statistical model, these values should be viewed as representing an estimate of the intrinsic “size” of leaves, as if each plant were harvested at the same developmental stage. Regardless of light treatment, Sum Length, Sum Width, and (Sum Area)^1/2^ varied extensively among accessions (**Fig. 3**).

The increase in leaf area we observe in wild tomato under simulated foliar shade conditions is at odds with what has previously been described in *Arabidopsis*, in which petiole length increases and blade area decreases under far-red treatments relative to white light [3]–[4], [13]. This discrepancy might be caused by differences in light treatment between different studies. However, it is also possible that wild tomato simply has an altered shade avoidance response with respect to leaf blade tissue compared to *Arabidopsis*.

**Figure 4:**
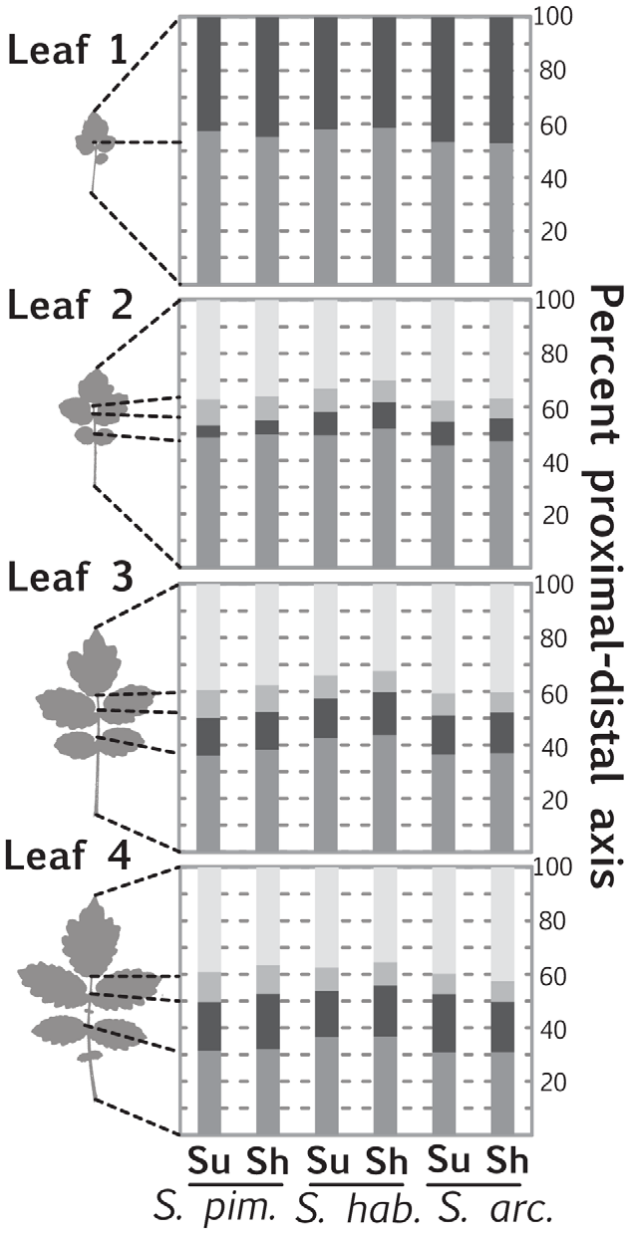
Elongation of the proximal-distal leaf axis in response to simulated foliar shade. Despite increases in leaf length in simulated shade treatment, the petiole region of tomato leaves does not expand at the expense of other parts of the leaf. Proportions of regions along the proximal-distal axis represent averaged accession values for each species. Regions measured include the terminal blade, the region between the terminal blade and oldest primary leaflets, the region between the oldest and youngest primary leaflets, and the petiole. Only terminal blade and the rest of the leaf 1 length were measured.

To investigate more fully the increase in wild tomato leaf length observed under simulated foliar conditions, we took advantage of the unique morphology of compound leaves. Previous literature in *Arabidopsis*, and a number of other species, has focused on petiole elongation in response to foliar shade, presumably because like internodes (the historical focus of shade avoidance response research) it is more stem-like than the expanded blade [2], [12], [23], [63]–[65]. We measured the proportion of the proximal-distal axis occupied by different regions of the tomato leaf under different light treatments: in leaf 1 we measured the proportion of the proximal-distal axis occupied by terminal blade versus the rest of the leaf; in leaves 2–4 we measured the extent of the proximal-distal axis occupied by terminal blade, the region from the terminal blade to the oldest primary leaflet, the region between the oldest and youngest primary leaflets, and the petiole (**Fig. 4**).

Based on the focus given to petiole elongation in the literature [4]–[5], we expected that the increase in leaf length observed in tomato leaves in simulated foliar conditions would be due to elongation of the petiole at the expense of the rest of the proximal-distal axis. To our surprise, the proportions occupied by different regions of the proximal-distal axis remained relatively constant between light treatments (**Fig. 4**). Such a result may still be consistent with the “petiole elongation” observed in *Arabidopsis*. Perhaps like tomato, *Arabidopsis* leaves also expand proportionally along all the regions of their proximal-distal axis, but because of the relatively vague petiole-blade boundary in *Arabidopsis* (in which the proximal blade region tapers into the petiole) decreases in blade area make it appear that the petiole region expands disproportionately. Such a hypothesis can be tested by examining whether or not expansion of cells in the midrib of the blade is similar to that in the petiole, separating the modulation of blade outgrowth from proximal-distal axis elongation.

**Figure 5:**
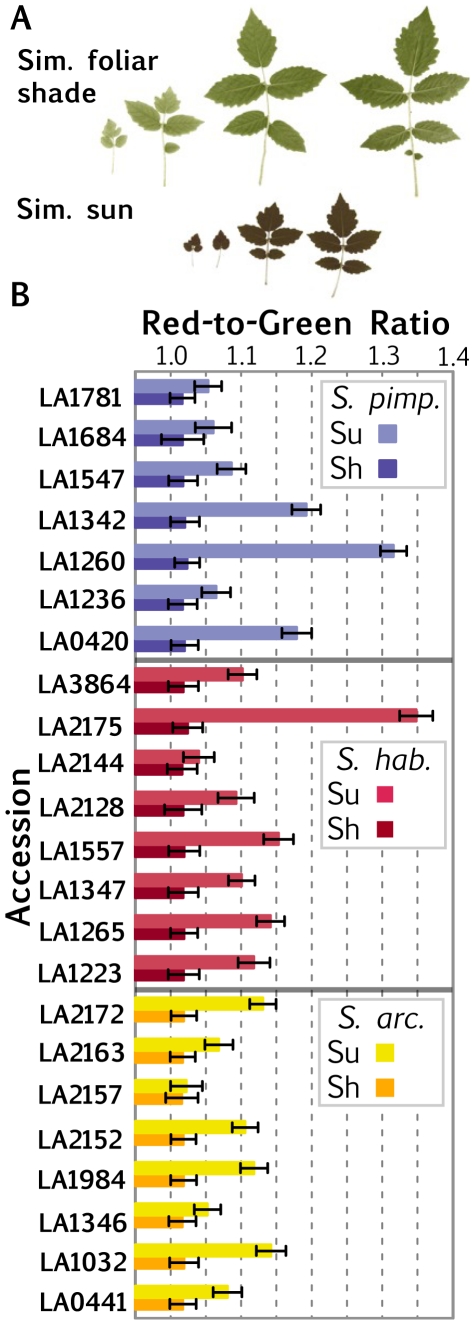
Colorimetry of shade avoidance in tomato leaves. A) Digital photographs of the abaxial side of leaf series taken under simulated shade and sun treatments. Note the accumulation of anthocyanins and other pigments that impart a dark, red color to leaves grown under simulated sun. B) R/G values of accessions calculated from RGB levels of the leaf series. Note that R/G ratios are nearly identical for all accession under simulated shade conditions, and that differences in the plasticity of this trait derive from differing R/G values under simulated sun. Blue, *S. pimpinellifolium*; Red, *S. habrochaites*; Yellow, *S. arcanum*. Darker shading, simulated shade; lighter shading, simulated sun. Bars represent SEM.

In addition to changes in leaf dimension, the shade avoidance response is also characterized by changes in pigment levels, including anthocyanins. Tomato differs from *Arabidopsis* in that its leaf blade is prominently purple-tinted on the abaxial side from the presence of anthocyanins [66] (**Fig. 5A**). Consistent with other species, leaves in tomato become paler in simulated foliar shade treatments from a decrease in chlorophyll and anthocyanin content [6]. We measured these pigment changes by proxy, using colorimetry of digital photographs taken under controlled conditions. From these photos we measured RGB levels of the abaxial side of leaves and calculated the Red-to-Green Ratio. Consistent with our qualitative observations, the Red-to-Green Ratio on the abaxial side of leaves decreased in simulated foliar shade treatment relative to simulated sun, reflecting the decrease in anthocyanin levels (**Fig. 5B**). Remarkably, the Red-to-Green Ratio between accessions is nearly constant under simulated foliar shade conditions, and variation in the plastic response of this trait is exclusively due to the differing Red-to-Green Ratio among accessions under simulated sun.

### Correlation of plastic leaf traits with NDVI

Most shade avoidance studies have focused on internode elongation and competition between plants along the stem axis as potentially adaptive traits [23], [67]–[68]. However, increase in blade tissue in response to foliar shade could also be feasibly adaptive, just as developmentally intrinsic leaf shape is thought to be an adaptive feature governed by water use efficiency. The lower leaf mass per area observed in shade avoiding plants may not only serve as a competitive mechanism to overgrow neighbors, but could also be an adaptive feature responding to the lower light intensity levels that usually accompany decreases in the red to far-red ratio of light [33], [69]–[70]. Light-responsive phenotypes have been found to correlate with latitude in *Arabidopsis* ecotypes, but whether this represents an adaptive response to light itself or another correlated factor remains to be determined [71]–[72]. Further, populations from exposed areas generally have more robust shade avoidance responses relative to those from wooded areas, presumably because a strong shade avoidance response in a heavily shaded region where escaping shade is not possible is maladaptive [25]–[29]. How shade avoidance responses might confer fitness advantages in the context of leaves and whether plastic leaf responses to enriched far-red light correlate with native levels of foliar shade is unknown.

**Figure 6:**
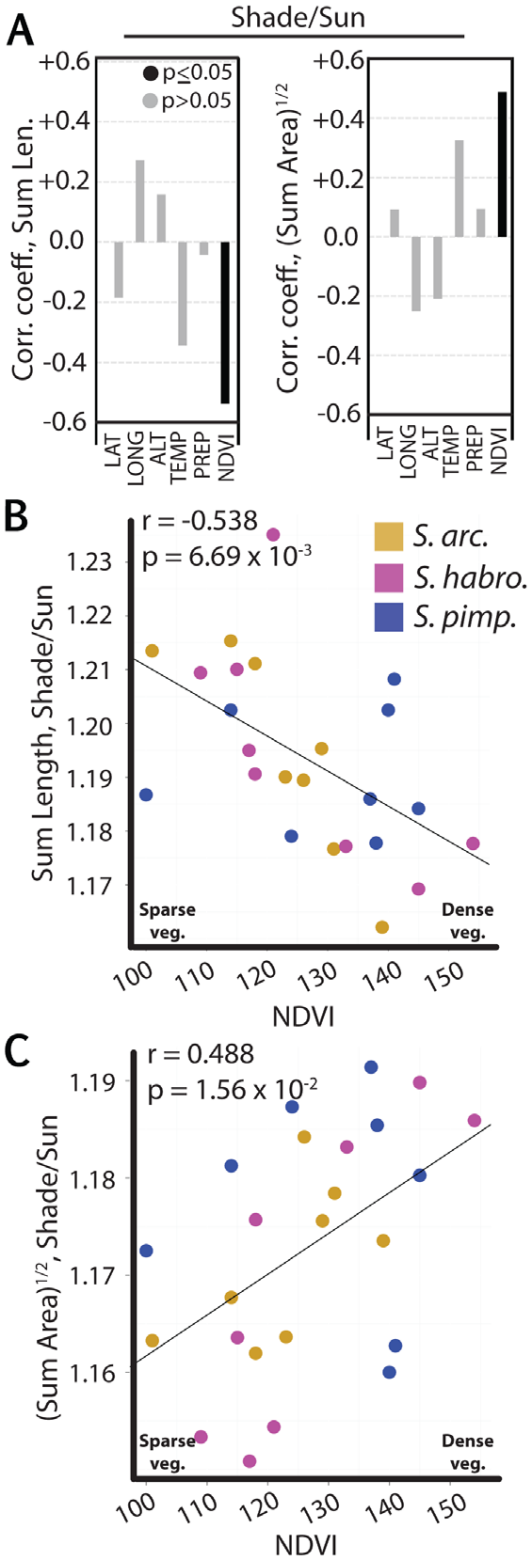
Correlation of plastic traits with environmental variables. *r* values (Pearson) representing correlations between the shade avoidance response in A) Sum Length and (Sum Area)^1/2^ of accessions with latitude, longitude, altitude, mean annual temperature, mean annual precipitation, and NDVI. Note that the plastic responses of Sum Length and (Sum Area)^1/2^ to simulated shade correlate with NDVI inversely, whereas both positively correlate with NDVI when only the simulated sun or shade treatment is considered (see **Fig. 2**). Scatter plots showing that the shade avoidance response for Sum Length (B) is negatively correlated with NDVI, whereas shade avoidance in (Sum Area)^1/2^ (C) is positively correlated with NDVI. Significance of *r* values deviating from 0 is denoted by solid (p<0.05) and gray (p>0.05) fill. *S. arcanum*, and *S. habrochaites*, and *S. pimpinellifolium* accessions are represented by gold, magenta, and navy, respectively.

To test for correlations between leaf plasticity and NDVI, we used the ratio of trait values under simulated foliar shade compared to simulated sun to measure the shade avoidance response. Shade avoidance in Sum Length is significantly negatively correlated with NDVI (**Fig. 6**). As a result of including Leaf Number in our models to adjust for developmental rate, such a relationship between shade avoidance and NDVI is anticipated. Because of the developmental rate correction, modeled changes in leaf dimension in response to foliar shade are fixed, and not proportional to the size of the leaf. In other words, all other factors being equal, smaller and larger leaves are constrained to increase in dimension to the same extent. To ask whether this is a reasonable assumption, we used the raw data to plot leaf length in simulated foliar shade as a function of length in simulated sun. This analysis of the raw data yields a linear model with a slope statistically indistinguishable from 1 (**Fig. S3**). If changes in leaf length in response to foliar shade had been proportional to leaf size, the slope would have been significantly greater than one. As a consequence of the response in leaf size to simulated shade being independent of leaf size, the ratio of leaf dimensions in simulated foliar shade to simulated sun necessarily decreases as leaf size increases. Because leaf length is positively correlated with NDVI (**Fig. 2**), it therefore follows that shade avoidance would be negatively correlated with NDVI (**Fig. 6**). These results suggest that intrinsic size of leaves indirectly impacts the relationship between plastic responses and the environment as a natural consequence of the way plants develop.

Strikingly, shade avoidance in (Sum Area)^1/2^ is positively correlated with NDVI values of accessions (**Fig. 6**). This result is surprising. If the leaf were a circle, and the length of a leaf equaled its width, we would expect mathematically that just as shade avoidance in leaf length is negatively correlated with NDVI, so too would be area. This relationship would even hold given a leaf shaped as an ellipse, on the condition that the length-to-width ratio of the ellipse remained constant between light treatments. That shade avoidance in (Sum Area)^1/2^ is positively correlated with NDVI suggests that one or more of these assumptions about leaf shape in tomato does not hold, and that there are aspects of leaf shape we do not account for in our model.

First, we consider whether the assumption of the constancy of the length-to-width ratio of leaves is violated. Fitting linear models of length against width for each leaf, changes in the length-to-width ratio between light treatments are apparent, but they are not large (**Fig. S7**). Using simple modeling, changes in leaf length-to-width ratio with respect to light treatment are not statistically supported (**Fig. S7E**). What is statistically supported is that the length-to-width ratio of leaves changes on a per leaf basis through the leaf series. Alone, this might not account for the discrepancies between shade avoidance in length and area that we observe; however, we are analyzing the sum of leaf lengths and areas across the leaf series. If the relative contribution of different leaves to that sum differs, this might contribute to the discrepancies we observe. Indeed, the relative sizes of leaves in the leaf series vary substantially for different accessions (**Fig. S8**). Furthermore, variation between accessions of length-to-width ratios across the leaf series exists (**Fig. S9**). Any number of these factors alone or in combination might contribute to the discrepancies between shade avoidance that we observe for Sum Length and (Sum Area)^1/2^.

Besides length-to-width ratios, the complexity of tomato leaves and the means by which they develop might also contribute to the anti-correlation between Sum Length and (Sum Area)^1/2^ shade avoidance responses. The leaves of tomato are far from simple circles or ellipses. Instead tomato leaves consist of a terminal leaflet at their distal end and a series of lateral leaflets that emerge from the rachis. The development of these leaves occurs basipetally (that is, growth originates from the proximal end of the leaf), and the terminal and distal lateral leaflets of a leaf are much larger than proximal lateral leaflets (see **Figs. 4** **,** **5A** **,** **7B** for visual examples). The differences in length-to-width ratio described above for leaves across the leaf series, and the relative contributions of different leaves to summed traits like Sum Length and (Sum Area)^1/2^, also pertain to leaflets along the proximal-distal axis of each leaf. Therefore, the differing directions of the correlations between shade avoidance response in dimension and area traits with NDVI likely represents to some degree the differential growth of laminar blade versus the proximal-distal axis of the leaf. Each of these in turn will also be affected by the plant's developmental stage.

**Figure 7:**
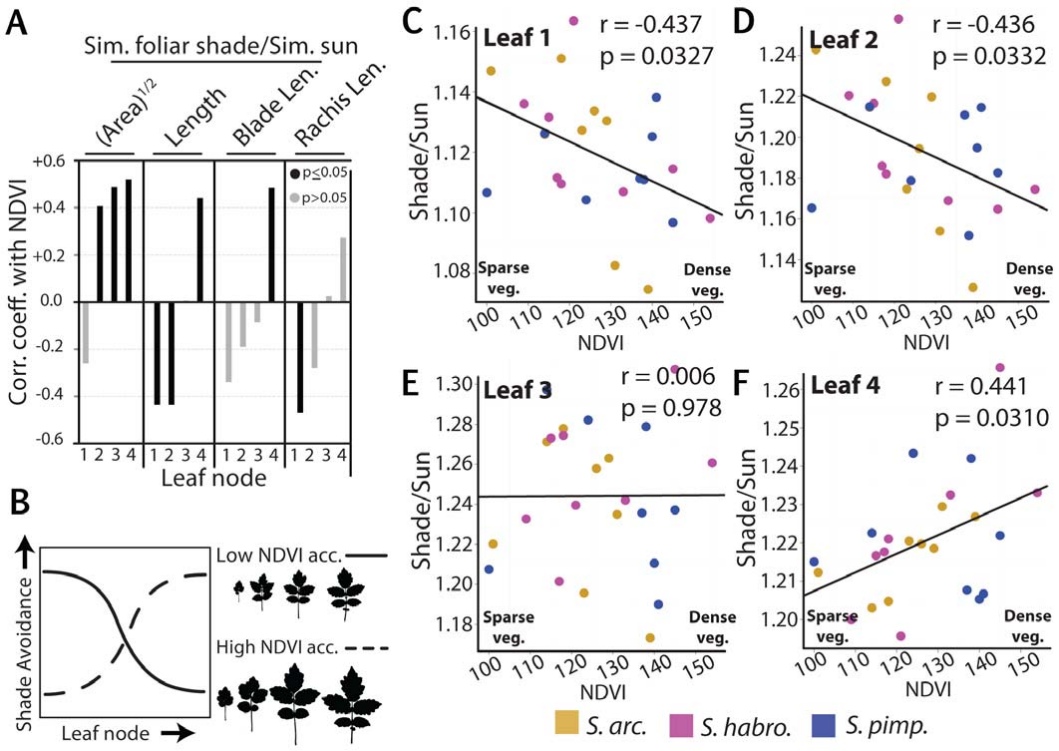
Correlation of shade avoidance response in individual leaves and sub-regions with NDVI. A) *r* values (Pearson) representing correlations between NDVI and plastic response values (Shade/Sun) for leaf area and length. Correlation between shade avoidance in sub-regions of the proximal-distal axis (terminal blade and rachis) and NDVI are given as well. Note that from leaf 1 to 4, the correlation for length begins significantly negative in leaf 1 and becomes significantly positive by leaf 4. Leaf area follows a similar trend. Correlations are stronger in blade regions of younger leaves and the rachis region of older leaves, suggesting an ontogenic component to shade avoidance. B) Cartoon representing the interpretation of results in (A) and the rest of this study. Regardless of light treatment, accessions from regions with low NDVI values (less foliar shade) have shorter leaves with smaller blade area relative to leaves from accessions with high NDVI values. The shade avoidance response in leaf length and area is greater in older leaves of accessions from regions of less foliar shade (solid line) and younger leaves of accessions from regions of high foliar shade. C–F) Shown are scatter plots representing the correlation of the plastic response to simulated shade in the length of each leaf with NDVI. Significance of *r* values deviating from 0 is denoted by solid (p<0.05) and gray (p>0.05) fill. *S. arcanum*, and *S. habrochaites*, and *S. pimpinellifolium* accessions are represented by gold, magenta, and navy, respectively.

Obviously, more sophisticated models of development are required in the future to fully discern relationships between leaf shape and size with the environment. What is apparent, however, is that the morphology of complex leaves can lead to discrepancies in the behavior of leaf blade area compared to leaf dimensions that might not occur in simple leaves with straightforward geometries.

### Modulation of shade avoidance across the leaf series

Sum Length, Sum Width, and (Sum Area)^1/2^ represent sums of values of the first four leaves of the leaf series. As shown in the previous section, these traits are an amalgamation of many different factors that vary between accessions, including: length-to-width ratios across the leaf series, the relative lengths of leaves in the series, and differences in how complex leaves are patterned (**Figs. S7, S8, S9**). How do the correlations with NDVI behave when analyzed for individual leaves?

The positive correlation observed for Sum Length and (Sum Area)^1/2^ traits under simulated foliar shade and simulated sun treatments with NDVI is displayed by individual leaves, although the correlation fails to achieve significance for leaf 1 area (**Fig. S10**). However, the behavior of plastic trait values across the leaf series is more complex. For shade avoidance in both length and area, there is a trend for the correlation with NDVI to begin negative and become positive in younger leaves (**Fig. 7**). For area, the first leaf has a non-significant negative correlation with NDVI and all the other leaves have significantly positive correlations, consistent with the overall positive correlation of shade avoidance response in (Sum Area)^1/2^ with NDVI (**Fig. 6**). Shade avoidance response in Sum Length, though, has a significantly negative correlation with NDVI, and this is true for the lengths of the first two leaves. However, the length of the third leaf is not correlated with NDVI, and by the fourth leaf shade avoidance response in length is positively correlated with NDVI (**Fig. 7**). The overall negative correlation between Sum Leaf Length shade avoidance and NDVI is consistent with this trait representing a sum (**Fig. S4**) and only one leaf of four showing positive correlation with NDVI.

The gradation, and eventual reversal, in the correlation of shade avoidance response of leaf length with NDVI across the leaf series is intriguing. It suggests that accessions from regions of low foliar shade have exaggerated shade avoidance phenotypes in their oldest—or first formed—leaves, whereas the opposite is true for accessions from regions with high foliar shade (**Fig. 7B**). An unequal distribution in shade avoidance along a developmental series has been previously described for internodes, where it was suggested selection might act to confine shade avoidance to discrete spatio-temporal contexts during development [28]. Superficially, this seems to be the case for leaf length in wild tomato accessions as well. How could this be achieved mechanistically?

One possibility is that individual leaves, throughout their development, would exhibit unique interactions between their shade avoidance phenotypes and native NDVI values. That is, each individual leaf would exhibit a distinct degree of shade avoidance throughout all developmental stages. This scenario is similar to that proposed earlier for internodes [28]. Such exquisite control of shade avoidance could be achieved through modulating genetic pathways regulating heteroblasty and/or juvenile-to-vegetative phase transition [73]–[74]. Early interpretations of shade leaf morphology, beginning with Goebel, reasoned that shade prolonged the juvenile phase of development [75]–[79].

It is also possible that leaves continuously alter the degree of their shade avoidance phenotypes during their ontogeny, and the differences in shade avoidance exhibited through the leaf series reflect that leaves farther in the series are younger than those at the beginning. Consistent with this idea, when the proximal-distal leaf axis was divided into lengths occupied by the terminal blade and the rachis-petiole, we found that these sub-regions mirrored the overall correlations of total leaf length plasticity with NDVI. However, it is interesting to note that the correlations are stronger in the terminal blade region for younger leaves, and in the rachis and petiole regions for older leaves (**Fig. 7A**). Also consistent with an ontogenic interpretation of this phenomenon is that the relative sizes of leaves in the series vary between accessions (**Fig. S8**). These size differences are a product of the developmental stage of the plant (as they change as the plant develops), and would therefore be reflective of ontogenic rather than heteroblastic processes.

Jones [80] noted that in *Cucurbita argyrospermia* the juvenile-appearing leaves of shaded plants were not the result of alterations in heteroblasty and the prolonging of juvenile development, but rather plastic responses of leaves to shade following a normal heteroblastic program. We favor a similar hypothesis, that the differences in shade avoidance response we observe throughout the tomato leaf series result from analyzing leaves at different stages in development. Generally leaves follow a prescribed developmental pattern of cell division that arrests in a basipetal fashion as the leaf matures [16], [81]. Cell enlargement processes follow a similar pattern, but are delayed relative to the wave of cell arrest that spreads through leaves. That young leaves have strong correlations in their shade avoidance response with NDVI in distal regions, whereas in older leaves the strongest correlations occur proximally, is reminiscent of the basipetal development of tomato leaves and reflects the regions of the leaf undergoing active growth. It might be that shade avoidance mechanisms act differentially upon early cell division processes versus later cell enlargement processes. However, it is also interesting to speculate whether shade avoidance response is mostly a phenomenon associated with actively growing regions and that differences in organ size attenuate as development ceases.

### Development and shade avoidance—interconnected, mutually informative processes

Our data demonstrate that shade avoidance response in tomato is characterized by increases in developmental rate, as measured by Leaf Number, and increases in Sum Length, Sum Width, and (Sum Area)^1/2^. However, shade avoidance response manifests itself distinctly in these different developmental processes. Populations from regions with higher NDVI values have lower plasticity to simulated foliar shade in terms of leaf number (**Table 1**). Sum Length and (Sum Area)^1/2^, contrastingly, are positively correlated with NDVI values within each light treatment, but are inversely correlated relative to NDVI with respect to their norms of reaction to simulated foliar shade (**Figs. 2** **,** **6**). The contrasting correlations in shade avoidance between Sum Length and (Sum Area)^1/2^ is likely caused by variation between accessions in the relative sizes of leaves in the series, their length-to-width ratios, and the differing morphology of leaflets and complexity of leaves. Further, the plastic responses of leaf dimensions and area across the leaf series are dynamic, such that shade avoidance responses are greater in older leaves of accessions from regions with less foliage, and exaggerated in younger leaves of accessions from regions with high foliar shade (**Fig. 7**). This likely reflects an ontogenic process related to the developmental stage of different leaves at any given time in the plant. What is abundantly clear is that shade avoidance manifests in complex ways as a natural consequence of how plants grow in relation to their environment. It remains to be shown whether the correlations we observe between traits and environmental factors are adaptive, but modeling and empirical evidence suggest that at least for leaf size, the maximization of water use efficiency relative to temperature and light intensity is an important factor.

What is apparent, however, is the exceptional degree to which development and shade avoidance are interconnected and can be mutually informative. Shade avoidance studies should consider separately developmental phenomena that are known to be controlled by unique genetic processes but influence, and potentially mask, responses of other traits to foliar shade (e.g., developmental rate). Even within a single organ, the leaf, shade avoidance traits across the leaf series correlate with NDVI in opposite directions as a downstream consequence of differences in developmental rate and how leaves differ between each other in the leaf series. Conversely, leaf development is traditionally studied as if occurring under “neutral” light conditions, and only recently have studies been undertaken to decipher how the shade avoidance response interacts with known leaf development pathways. Undoubtedly, the levels of foliar shade from where a population originates have led to adjustments in development that are not considered during most analyses. Outside of paleobotanical and ecological studies, little has been done to investigate how different attributes of leaves—such as their size, shape, and complexity—are potentially adaptive. Studying wild accessions of species, which provide an ample diversity of leaf morphology traits, under controlled environmental conditions that reveal potentially adaptive norms of reaction, can be exploited for association mapping studies in the future to discover genetic natural variation regulating biologically relevant developmental processes.

## Supporting Information

Figure S1 **Shade avoidance response of developmental rate.** Trait values of accessions for Leaf Number (LFN) derived from mixed-effect linear models. There is a significant increase in LFN under simulated shade treatment relative to simulated sun. Blue, *S. pimpinellifolium*; Red, *S. habrochaites*; Yellow, *S. arcanum*. Darker shading, simulated shade; ligher shading, simulated sun. Bars represent SEM.
(TIF)

Figure S2 **Leaf number is correlated with other traits.** Plots of A) Sum Length, B) Sum Width, and C) (Sum Area)^1/2^ against ln(leaf number (LFN)) for all measured data points. Significant, positive correlations are observed because plants with higher LFN values develop faster and produce larger leaves than those with smaller LFN values that develop more slowly. ln(LFN) was used to derive mixed-effect linear models because of its more linear relationship with trait values compared to LFN.
(TIF)

Figure S3 **Increases in leaf size in response to simulated shade are not proportional to leaf size.** Scatter plot showing means of Sum Length taken from raw data of accessions under simulated shade versus simulated sun conditions. Because we use Leaf Number (LFN) to correct for developmental rate in our models, modeled increases in leaf dimension in response to simulated foliar shade are not proportional to intrinsic leaf size; that is, small and large leaves will all increase their dimensions by the same amount in response to foliar shade. This assumption is supported in the raw data shown here, in that the slope of a linear model plotting leaf length in simulated shade vs. sun is not statistically distinguishable from a slope equal to 1 (p = 0.23). Blue, fitted linear model; dotted gray, line y = x.
(TIF)

Figure S4 **Diagrammatic representation of traits measured in this study.** Sum Length, Sum Width, and Sum Area represent the sums of the respective measures across the first four leaves. Leaflet Area is the averaged areas of the two most distal leaflets of leaf 3.
(TIF)

Figure S5 **Principal Component Analysis (PCA) on highly correlated leaf dimension traits.** A) Because of the high correlation between leaf dimension traits (**Fig. 3**), a PCA was performed on Sum Length, Sum Width, (Sum Area)^1/2^, and (Lft. Area)^1/2^. In the PCA, each accession is represented twice: once for its leaf dimension values under simulated sun and once for its leaf dimension values under simulated shade. The two points for each accession are connected by an arrow, with the base of the arrow representing simulated sun data and the tip simulated shade data. Percent variation explained by PC1 and PC2 is indicated. B) Correlations (*r* value, Pearson) between PC1 values under simulated sun and simulated shade with environmental variables is shown. Shade avoidance was approximated as the Euclidian distance (calculated for PCs 1–4) between simulated sun and shade. *r* values in bold are statistically significant. Correlations correspond with those shown through conventional means in **Fig. 2**. *S. arcanum*, *S. habrochaites*, and *S. pimpinellifolium* accessions are represented by gold, magenta, and navy, respectively. *p<0.05, **p<0.01, ***p<0.001.
(TIF)

Figure S6 **Shade avoidance response in PSQA values.** Perimeter/(area)^1/2^ (PSQA) trait values of accessions for A) leaves and B) leaflets. Although treatment is a significant factor in the mixed-effect linear models, the difference in PSQA values between light conditions is slight. In leaves, PSQA is slightly higher under simulated shade conditions (most obvious in accessions with overall low PSQA values). In leaflets, PSQA is slightly higher under simulated sun conditions (most obvious in accessions with overall high PSQA values). These differences between treatments in PSQA may reflect increased complexity in leaves under low simulated shade conditions and increased serration under simulated sun conditions. Further analyses in more mature leaves is needed to confirm these hypotheses. Blue, *S. pimpinellifolium*; Red, *S. habrochaites*; Yellow, *S. arcanum*. Darker shading, simulated shade; lighter shading, simulated sun. Bars represent SEM.
(TIF)

Figure S7 **Length-to-width ratio changes are observed across the leaf series and not between light treatments.** A–D) Mean leaf length versus width for individual leaves in the leaf series. X and Y axes are at the same scale between panels. Fitted linear models in blue and orange represent simulated shade and sun data, respectively. E) Changes in length-to-width ratio are indicated by significant changes in slope. An ANCOVA model of length as a function of width, treatment, leaf node, and interaction terms supports that the length-to-width ratio of leaves changes between leaves in the series. Changes in the length-to-width ratio of leaves between light treatments is not supported.
(TIF)

Figure S8 **Variation in leaf dimensions across the leaf series amongst accessions.** Line graphs for mean leaf A) length, B) width, and C) area values across the leaf series for accessions under simulated sun and shade treatments. Values across the series have been mean-centered at zero, to better reflect changes in the pattern of leaf size across the series rather than overall differences in size. Although there is an overall trend of increasing leaf size through the series, accessions vary as to which leaves in the series are most prominent. Together with the differences in length-to-width ratio observed in leaves at different nodes (**Fig. S7**), these data may explain the differing correlations in shade avoidance of Sum Length and (Sum Area)^1/2^ observed with NDVI (as discussed in the text and **Fig. 6**). *S. arcanum*, *S. habrochaites*, and *S. pimpinellifolium* accessions are represented by gold, magenta, and navy, respectively.
(TIF)

Figure S9 **Differences between accessions in the length-to-width ratio of leaves.** Mean length-to-width ratio versus leaf node under simulated sun and shade conditions for different accessions. *S. arcanum*, and *S. habrochaites*, and *S. pimpinellifolium* accessions are represented by gold, magenta, and navy, respectively.
(TIF)

Figure S10 **Positive correlation between leaf traits and NDVI across the leaf series.** *r* values representing correlation between leaf dimension traits and sub-regions of the length of the proximal-distal axis with NDVI under A) simulated sun and B) simulated shade conditions. Significance of *r* values deviating from 0 is denoted by solid (p<0.05) and gray (p>0.05) fill.
(TIF)

Table S1 **p-values for significant terms in the mixed-effect linear models used in this study.** Please see Material & Methods in text for details.
(PDF)

Table S2 **Attributes of accessions and inter-correlation.** A) Table of latitude and longitude (degrees), altitude (m), mean annual precipitation (mm), mean annual temperature (°C*10), and NDVI values for accessions used in this study. *S. arcanum* (yellow), *S. habrochaites* (red), and *S. pimpinellifolium* (blue) accessions are denoted by their respective colors. B) Table showing correlation coefficients between environmental variables. Significant correlations are highlighted in yellow.
(TIF)

## References

[1] Vandenbussche F, Pierik R, Millenaar FF, Voesenek LA, Van Der Straeten D (2005). Reaching out of the shade.. Curr Opin Plant Biol.

[2] Pierik R, Cuppens ML, Voesenek LA, Visser EJ (2004). Interactions between ethylene and gibberellins in phytochrome-mediated shade avoidance responses in tobacco.. Plant Physiol.

[3] Kozuka T, Horiguchi G, Kim GT, Ohgishi M, Sakai T (2005). The different growth responses of the *Arabidopsis thaliana* leaf blade and petiole during shade avoidance are regulated by photoreceptors and sugar.. Plant Cell Physiol.

[4] Kim GT, Yano S, Kozuka T, Tsukaya H (2005). Photomorphogenesis of leaves: shade-avoidance and differentiation of sun and shade leaves.. Photochem Photobiol Sci.

[5] Franklin KA (2008). Shade avoidance.. New Phytol.

[6] Smith H, Whitelam GC (1997). The shade avoidance syndrome: multiple responses mediated by multiple phytochromes.. Plant, Cell, Environ.

[7] Neff MM, Frankhauser C, Chory J (2000). Light: an indicator of time and place.. Genes Dev.

[8] Endo M, Nakamura S, Araki T, Mochizuki N, Nagatani A (2005). Phytochrome B in the mesophyll delays flowering by suppressing FLOWERING LOCUS T expression in Arabidopsis vascular bundles.. Plant Cell.

[9] Kozuka T, Kobayashi J, Horiguchi G, Demura T, Sakakibara H (2010). Involvement of auxin and brassinosteroid in the regulation of petiole elongation under shade.. Plant Physiol.

[10] Nagatani A, Chory J, Furuya M (1991). Phytochrome B is not detectable in the *hy3* mutant of Arabidopsis, which is deficient in responding to end-of-day far-red light treatments.. Plant Cell Physiol.

[11] Reed JW, Nagpal P, Poole DS, Furuya M, Chory J (1993). Mutations in the gene for the red/far-red light receptor phytochrome B alter cell elongation and physiological responses throughout Arabidopsis development.. Plant Cell.

[12] Robson PR, Whitelam GC, Smith H (1993). Selected components of the shade-avoidance syndrome are displayed in a normal manner in mutants of Arabidopsis thaliana and Brassica rapa deficient in phytochrome B.. Plant Physiol.

[13] Tsukaya H, Kozuka T, Kim GT (2002). Genetic control of petiole length in *Arabidopsis thaliana*.. Plant Cell Physiol.

[14] Whitelam GC, Delvin PF (1997). Roles for different phytochromes in *Arabidopsis* photomorphogenesis.. Plant Cell Environ.

[15] Avery GS (1932). Structure and development of the tobacco leaf.. Amer J Bot.

[16] Donnelly PF, Bonetta D, Tsukaya H, Dengler RE, Dengler NG (1999). Cell cycling and cell enlargement in developing leaves of *Arabidopsis*.. Dev Biol.

[17] Tsuge T, Tsukaya H, Uchimiya H (1996). Two independent and polarized processes of cell elongation regulate leaf blade expansion in *Arabidopsis thaliana* (L.) Heynh.. Development.

[18] Kim GT, Shoda K, Tsuge T, Sho KH, Uchimiya H (2002). The *ANGUSTIFOLIA* gene of *Arabidopsis*, a plant *CtBP* gene, regulates leaf-cell expansion, the arrangement of microtubules in leaf cells and expression of a gene involved in cell wall formation.. EMBO J.

[19] Kim GT, Tsukaya H, Uchimiya H (1998). The *ROTUNDIFOLIA3* gene of *Arabidopsis thaliana* encodes a new member of the cytochrome P450 family that is required for the regulated polar elongation of leaf cells.. Genes Dev.

[20] Kim GT, Fujioka S, Kozuka T, Tax FE, Takatsuto S (2005). CYP90C1 and CYP90D1 are involved in different steps in the brassinosteroid biosynthesis pathway in Arabidopsis thaliana.. Plant J.

[21] Sakamoto T, Kamiya N, Ueguchi-Tanaka M, Iwahori S, Matsuoka M (2001). KNOX homeodomain protein directly suppresses the expression of a gibberellin biosynthetic gene in the tobacco shoot apical meristem.. Genes Dev.

[22] Pierik R, Whitelam GC, Voesenek LA, deKroon H, Visser EJ (2004). Canopy studies on ethylene-insensitive tobacco identify ethylene as a novel element in blue light and plant-plant signaling.. Plant J.

[23] Djakovic-Petrovic T, de Wit M, Voesenek LA, Perik R (2007). DELLA protein function in growth responses to canopy signals.. Plant J.

[24] Hisamatsu T, King RW, Helliwell CA, Koshioka M (2005). The involvement of gibberellin 20-oxidase genes in phytochrome-regulated petiole elongation of Arabidopsis.. Plant Physiol.

[25] Morgan DC, Smith H (1979). A systematic relationship between phytochrome-controlled development and species habitat, for plants grown in simulated natural radiation.. Planta.

[26] Bain AB, Attridge TH (1988). Shade-light mediated responses in field and hedgerow populations of *Galium aparine* L.. Journal of Experimental Botany.

[27] Van Tienderen PH, Van Hinsberg A (1996). Phenotypic plasticity in growth habit in *Plantago lanceolata*: how tight is a suite of correlated characters?. Plant Species Biology.

[28] Dudley S, Schmitt J (1995). Genetic differentiation between open and woodland *Impatiens capensis* populations in morphological responses to simulated foliage shade.. Functional Ecology.

[29] Schmitt J (1997). Is photomorphogenic shade avoidance adaptive? Perspectives from population biology.. Plant, Cell, and Environment.

[30] Sawers RJ, Sheehan MJ, Brutnell TP (2005). Cereal phytochromes: targets of selection, targets for manipulation?. Trends Plant Sci.

[31] Sellers PJ (1985). Canopy reflectance, photosynthesis, and transpiration.. International Journal of Remote Sensing.

[32] Myneni RB, Hall FG, Sellers PJ, Marshak AL (1995). The interpretation of spectral vegetation indexes.. IEEE Transactions on Geoscience and Remote Sensing.

[33] Poorter H, Niinemets U, Walter A, Fiorani F, Schurr U (2010). A method to construct dose-response curves for a wide range of environmental factors and plant traits by means of meta-analysis of phenotypic data.. Journal of Experimental Botany.

[34] Brown WH (1919). Vegetation of the Philippine Islands.. Philippine Bureau of Science, Dep of Agric & Natural Res (Manila).

[35] Cain SA, Castro GM, de Oliviera, Pires JM, da Silva NT (1956). Application of some phytosociological techniques to Brazilian rain forest.. Am J Bot.

[36] Parkhurst DF, Loucks OL (1972). Optimal leaf size in relation to environment.. Journal of Ecology.

[37] Givnish TJ, Vermeij GJ (1976). Sizes and shapes of liane leaves.. The American Naturalist.

[38] Nicotra AB, Leigh A, Boyce CK, Jones CS, Niklas KJ (2011). The evolution and functional significance of leaf shape in the angiosperms.. Functional Plant Biology.

[39] Clements ES (1904). The relation of leaf structure to physical factors.. Trans Am Microsc Soc.

[40] Graner EA (1942). Genetics of Manihot. I. Inheritance of leaf form and color of the outer root skin in Manihot utilissima Pohl.. Bragantia.

[41] Shields LM (1950). Leaf xeromorphy as related to physiological and structural influences.. Bot Rev.

[42] Blackman GE, Milthorpe FL (1956). Influence of light and temperature on leaf growth.. The Growth of Leaves.

[43] Talbert CM, Holch AE (1957). A study of the lobing of sun and shade leaves.. Ecology.

[44] Richards PW (1964). The Tropical Rain Forest.

[45] Bailey IW, Sinnott EW (1915). A botanical index of Cretaceous and Tertiary climates.. Science.

[46] Wolfe JA (1971). Tertiary climate fluctuations and methods of analysis of Tertiary floras.. Palaeogeography, Palaeoclimatology, Palaeoecology.

[47] Greenwood DR (1992). Taphonomic constraints on foliar physiognomic interpretations of Late Cretaceous and Tertiary palaeoclimates.. Review of Palaeobotany and Palynology.

[48] Carpenter RJ, Hill RS, Jordan GJ, Hill RS (1994). Cenozoic vegetation in Tasmania: macrofossil evidence.. History of the Australian vegetation: Cretaceous to Recent.

[49] Dilcher DL, Graham A (1973). A paleoclimatic interpretation of the Eocene floras of southeastern North America.. Vegetation and vegetational history of northern Latin America.

[50] Wilf P, Wing SL, Greenwood DR, Greenwood CL (1998). Using fossil leaves as paleoprecipitation indicators: an Eocene example.. Geology.

[51] Webb LJ (1968). Environmental relationships of the structural types of Australian rain forest vegetation.. Ecology.

[52] Dolph GE, Dilcher DL (1980). Variation in leaf size with respect to climate in Costa Rica.. Biotropica.

[53] Dolph GE, Dilcher DL (1980). Variation in leaf size with respect to climate in the tropics of the Western Hemisphere.. Bulletin of the Torrey Botanical Club.

[54] Royer DL, McElwain JC, Adams JM, Wilf P (2008). Sensitivity of leaf size and shape to climate within *Acer rubrum* and *Quercus kelloggii*.. New Phytologist.

[55] Royer DL, Meyerson LA, Robertson KM, Adams JM (2009). Phenotypic plasticity of leaf shape along a temperature gradient in *Acer rubrum*.. PLoS One.

[56] Moyle LC (2008). Ecological and evolutionary genomics in the wild tomatoes (*Solanum* Sect. *Lycopersicon*).. Evolution.

[57] Nakazato T, Warren DL, Moyle LC (2010). Ecological and geographic modes of species divergence in wild tomatoes.. American Journal of Botany.

[58] Rasband WS (1997–2011). ImageJ.. http://imagej.nih.gov/ij/.

[59] Bylesjö M, Segura V, Soolanayakanahally RY, Rae AM, Trygg J (2008). LAMINA: a tool for rapid quantification of leaf size and shape parameters.. BMC Plant Biology.

[60] R Development Core Team (2009). R: A language and environment for statistical computing.. http://www.R-project.org.

[61] Bates D, Maechler M (2009). lme4: Linear mixed-effects models using S4 classes.. http://CRAN.Rproject.org/package=lme4.

[62] Peralta IE, Spooner DM, Knapp S (2008). Taxonomy of wild tomatoes and their relatives (Solanum sect. Lycopersicoides, sect. Juglandifolia, sect. Lycopersicon Solanaceae).. Syst Bot Monogr.

[63] Morgan DC, Smith H (1976). Linear relationship between phytochrome photoequilibrium and growth in plants under simulated natural radiation.. Nature.

[64] Morgan DC, Smith H (1978). The function of phytochrome in the natural environment. VII The relationship between phytochrome photo-equilibrium and development in light-grown *Chenopodium album* L.. Planta.

[65] Morgan DC, Smith H (1981). Control of development in *Chenopodium album* L. by shadelight—the effect of light quantity (total fluence rate) and light quality (red:far-red ratio).. New Phytologist.

[66] Weller JL, Schreuder ME, Smith H, Koornneef M, Kendrick RE (2000). Physiological interactions of phytochromes A, B1, and B2 in the control of development in tomato.. Plant J.

[67] Donohue K, Messiqua D, Pyle EH, Heschel MS, Schmitt J (2000). Evidence of adaptive divergence in plasticity: density- and site-dependent selection on shade avoidance responses in *Impatiens capensis*.. Evolution.

[68] Schmitt J, Stinchcombe JR, Heschel MS, Huber H (2003). The adaptive evolution of plasticity: phytochrome-mediated shade avoidance responses.. Integr Comp Biol.

[69] Maliakel SK, McDonnell K, Dudley SA, Schmitt J (1999). Effects of red to far-red ratio and plant density on biomass allocation and gas exchange in *Impatiens capensis*.. International J Plant Sci.

[70] Boccalandro HE, Rugnone ML, Moreno JE, Ploschuk EL, Serna L (2009). Phytochrome B enhances photosynthesis at the expense of water-use efficiency in Arabidopsis.. Plant Physiol.

[71] Maloof JN, Borevitz JO, Dubi T, Lutes J, Nehring RB (2001). Natural variation in light sensitivity in Arabidopsis.. Nature Genetics.

[72] Balusbramanian S, Sureshkumar S, Agrawal M, Michael TP, Wessinger C (2006). The PHYTOCHROME C photoreceptor gene mediates natural variation in flowering and growth responses of Arabidopsis thaliana.. Nat Genet.

[73] Kerstetter RA, Poethig RS (1998). The specification of leaf identity during shoot development.. Annu Rev Cell Dev Biol.

[74] Poethig RS (2003). Phase change and the regulation of developmental timing in plants.. Science.

[75] Goebel K (1908). Einleitung in die experimentelle Morphologie der Pflanzen..

[76] Ashby E (1948). Studies in the morphogenesis of leaves. I. An essay on leaf shape.. New Phytologist.

[77] Njoku E (1956). Studies in the morphogenesis of leaves. XI. The effect of light intensity on leaf shape in *Ipomoea caerulea*.. New Phytologist.

[78] Montaldi ER, Caso OH, Lewin IJ (1963). Algunos factores que afectan la morfologia de las hojas en una planta de desarrollo heteroblastico.. Revisita de Investigaciones Agricoles (Buenos Aires).

[79] Cameron RJ (1970). Light intensity and the growth of Eucalyptus seedlings. I. Ontogenic variation in E. fastigata.. Australian Journal of Botany.

[80] Jones CS (1995). Does shade prolong juvenile development? A morphological analysis of leaf shape changes in Cucurbita argyrosperma Subsp. Sororia (Curcubitaceae).. American Journal of Botany.

[81] Kang J, Dengler N (2002). Cell cycling frequency and expression of the homeobox gene ATHB8 during leaf vein development in Arabidopsis.. Planta.

