# Supplementary figures and images for: Native Environment Modulates Leaf Size and Response to Simulated Foliar Shade across Wild Tomato Species
Source: PLoS One. 2012 Jan 12;7(1):e29570. doi: 10.1371/journal.pone.0029570 (PMC3257252; doi:10.1371/journal.pone.0029570)

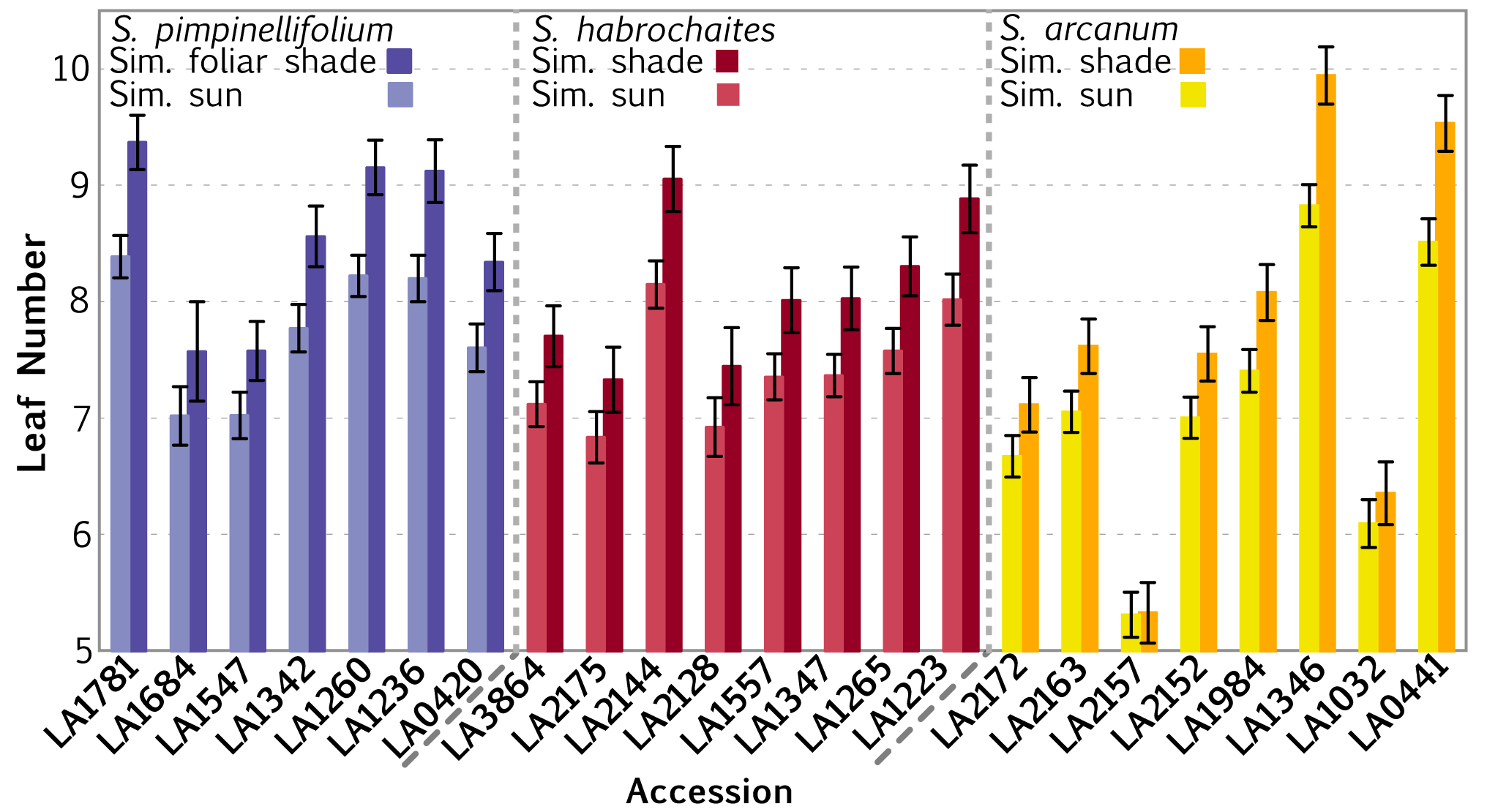

Supplement: Figure S1 — Shade avoidance response of developmental rate. Trait values of accessions for Leaf Number (LFN) derived from mixed-effect linear models. There is a significant increase in LFN under simulated shade treatment relative to simulated sun. Blue, S. pimpinellifolium; Red, S. habrochaites; Yellow, S. arcanum. Darker shading, simulated shade; ligher shading, simulated sun. Bars represent SEM. (TIF) [file pone.0029570.s001.tif]

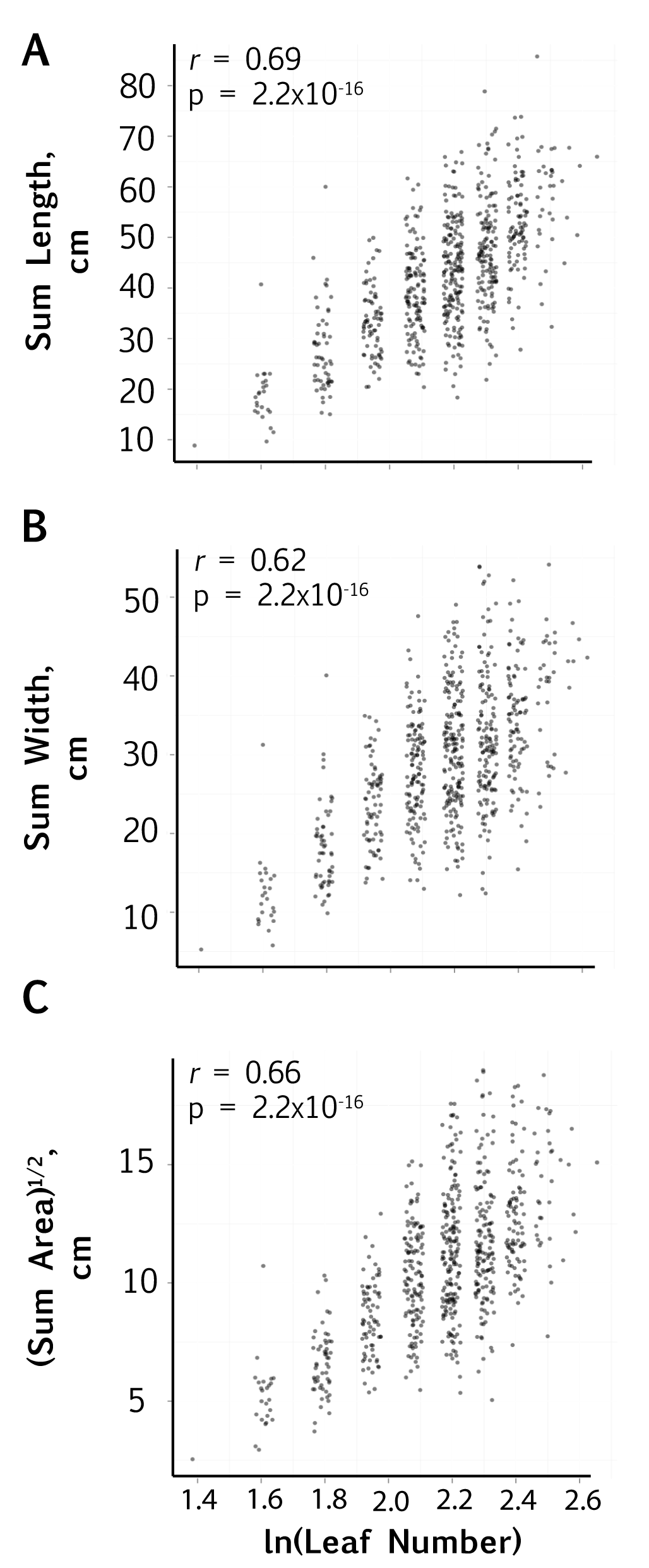

Supplement: Figure S2 — Leaf number is correlated with other traits. Plots of A) Sum Length, B) Sum Width, and C) (Sum Area)1/2 against ln(leaf number (LFN)) for all measured data points. Significant, positive correlations are observed because plants with higher LFN values develop faster and produce larger leaves than those with smaller LFN values that develop more slowly. ln(LFN) was used to derive mixed-effect linear models because of its more linear relationship with trait values compared to LFN. (TIF) [file pone.0029570.s002.tif]

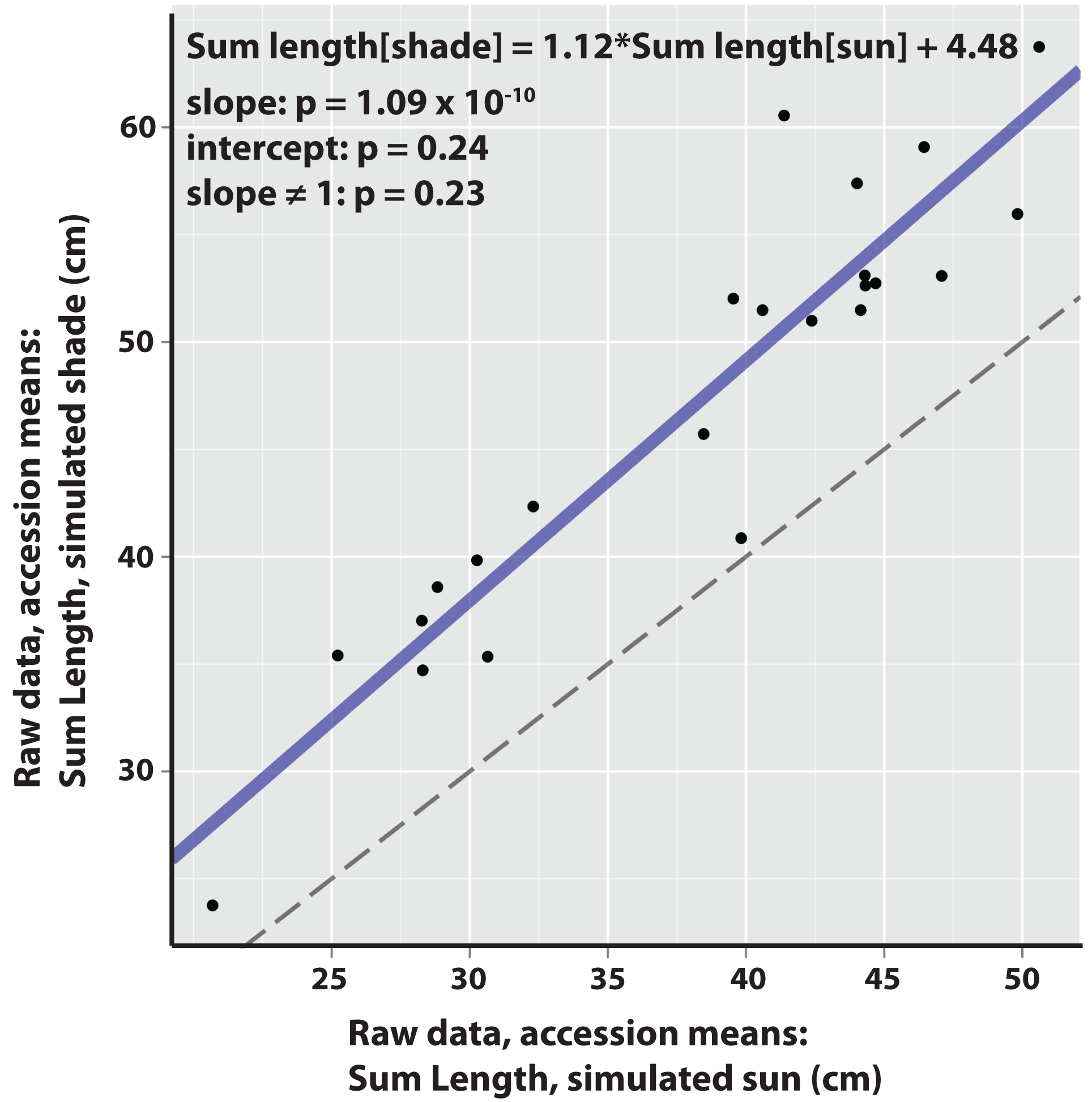

Supplement: Figure S3 — Increases in leaf size in response to simulated shade are not proportional to leaf size. Scatter plot showing means of Sum Length taken from raw data of accessions under simulated shade versus simulated sun conditions. Because we use Leaf Number (LFN) to correct for developmental rate in our models, modeled increases in leaf dimension in response to simulated foliar shade are not proportional to intrinsic leaf size; that is, small and large leaves will all increase their dimensions by the same amount in response to foliar shade. This assumption is supported in the raw data shown here, in that the slope of a linear model plotting leaf length in simulated shade vs. sun is not statistically distinguishable from a slope equal to 1 (p = 0.23). Blue, fitted linear model; dotted gray, line y = x. (TIF) [file pone.0029570.s003.tif]

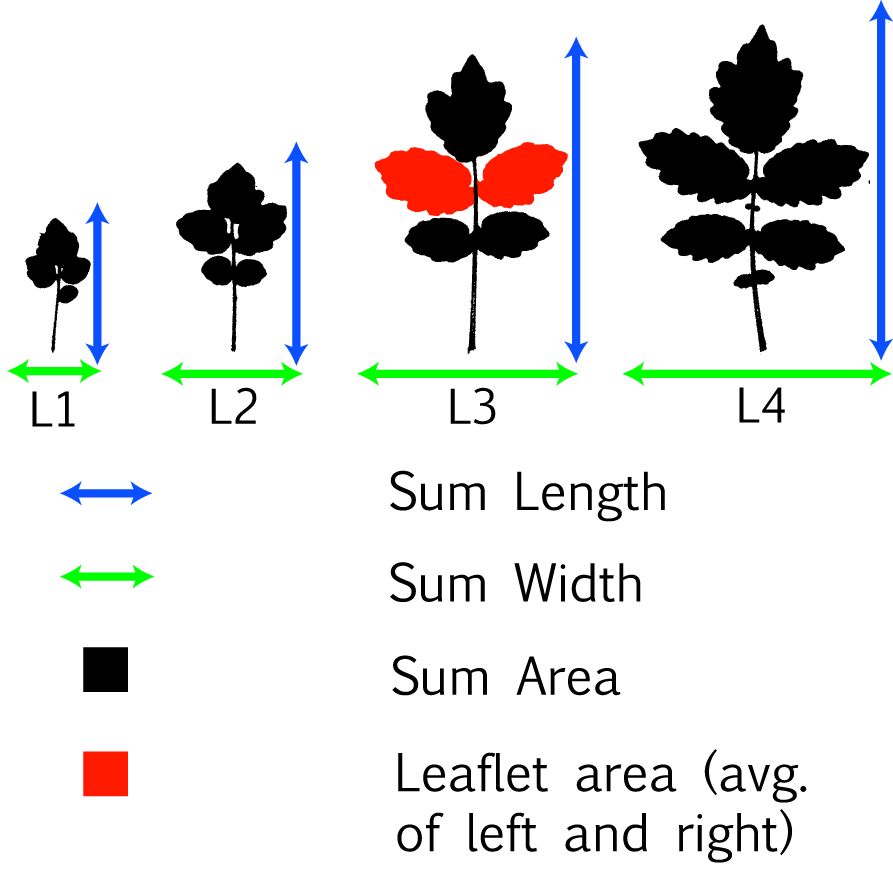

Supplement: Figure S4 — Diagrammatic representation of traits measured in this study. Sum Length, Sum Width, and Sum Area represent the sums of the respective measures across the first four leaves. Leaflet Area is the averaged areas of the two most distal leaflets of leaf 3. (TIF) [file pone.0029570.s004.tif]

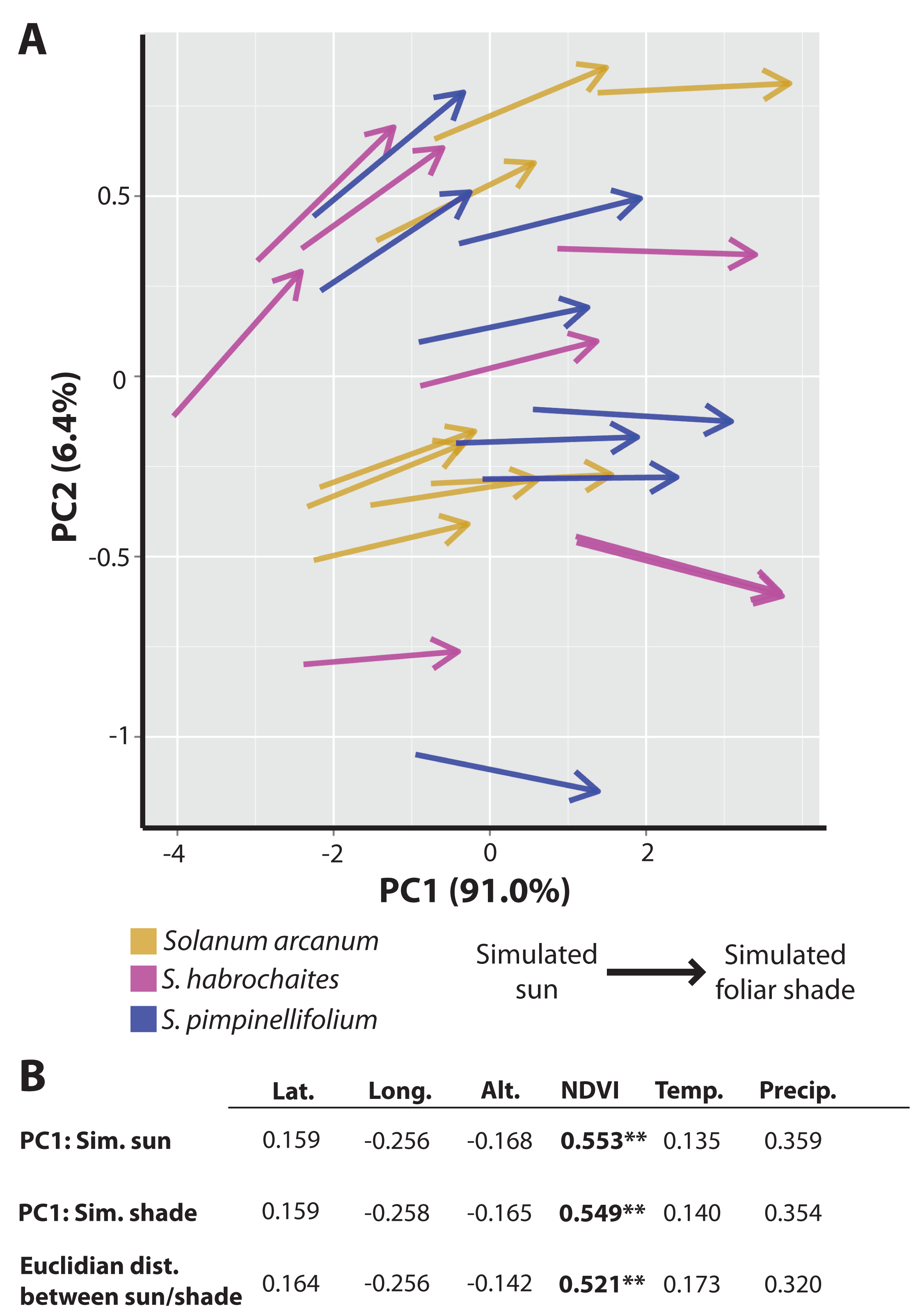

Supplement: Figure S5 — Principal Component Analysis (PCA) on highly correlated leaf dimension traits. A) Because of the high correlation between leaf dimension traits ( Fig. 3 ), a PCA was performed on Sum Length, Sum Width, (Sum Area)1/2, and (Lft. Area)1/2. In the PCA, each accession is represented twice: once for its leaf dimension values under simulated sun and once for its leaf dimension values under simulated shade. The two points for each accession are connected by an arrow, with the base of the arrow representing simulated sun data and the tip simulated shade data. Percent variation explained by PC1 and PC2 is indicated. B) Correlations (r value, Pearson) between PC1 values under simulated sun and simulated shade with environmental variables is shown. Shade avoidance was approximated as the Euclidian distance (calculated for PCs 1–4) between simulated sun and shade. r values in bold are statistically significant. Correlations correspond with those shown through conventional means in Fig. 2 . S. arcanum, S. habrochaites, and S. pimpinellifolium accessions are represented by gold, magenta, and navy, respectively. *p<0.05, **p<0.01, ***p<0.001. (TIF) [file pone.0029570.s005.tif]

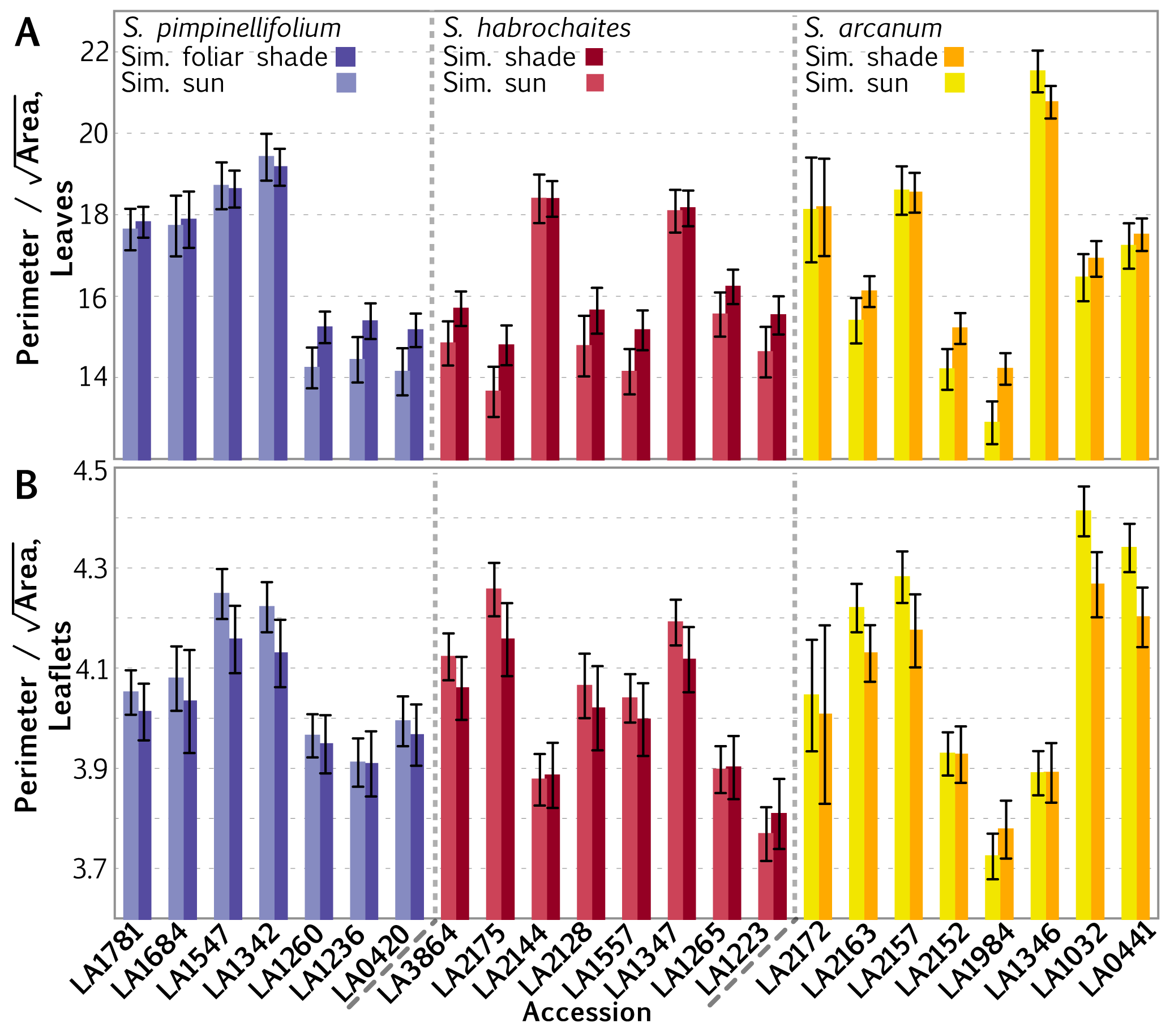

Supplement: Figure S6 — Shade avoidance response in PSQA values. Perimeter/(area)1/2 (PSQA) trait values of accessions for A) leaves and B) leaflets. Although treatment is a significant factor in the mixed-effect linear models, the difference in PSQA values between light conditions is slight. In leaves, PSQA is slightly higher under simulated shade conditions (most obvious in accessions with overall low PSQA values). In leaflets, PSQA is slightly higher under simulated sun conditions (most obvious in accessions with overall high PSQA values). These differences between treatments in PSQA may reflect increased complexity in leaves under low simulated shade conditions and increased serration under simulated sun conditions. Further analyses in more mature leaves is needed to confirm these hypotheses. Blue, S. pimpinellifolium; Red, S. habrochaites; Yellow, S. arcanum. Darker shading, simulated shade; lighter shading, simulated sun. Bars represent SEM. (TIF) [file pone.0029570.s006.tif]

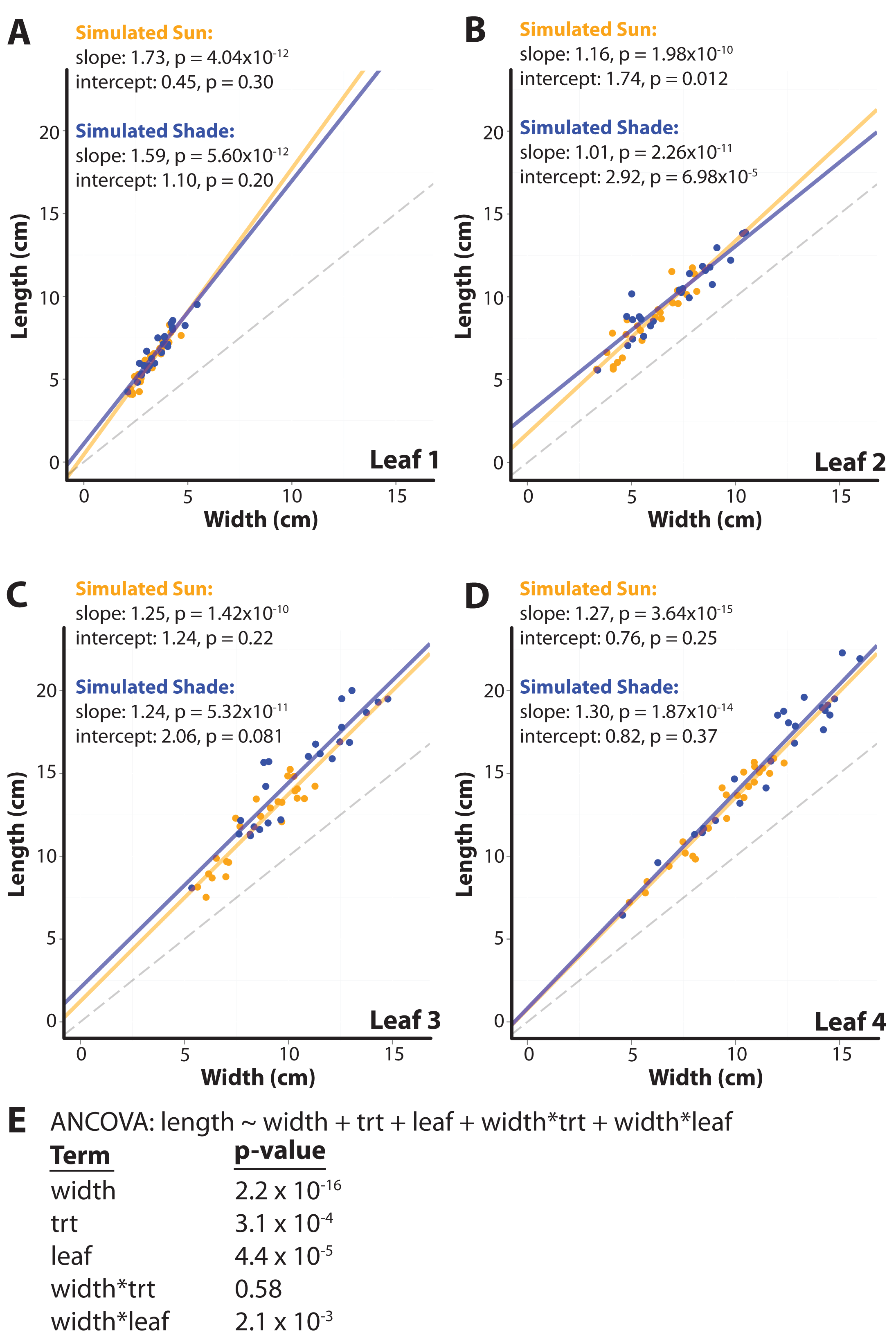

Supplement: Figure S7 — Length-to-width ratio changes are observed across the leaf series and not between light treatments. A–D) Mean leaf length versus width for individual leaves in the leaf series. X and Y axes are at the same scale between panels. Fitted linear models in blue and orange represent simulated shade and sun data, respectively. E) Changes in length-to-width ratio are indicated by significant changes in slope. An ANCOVA model of length as a function of width, treatment, leaf node, and interaction terms supports that the length-to-width ratio of leaves changes between leaves in the series. Changes in the length-to-width ratio of leaves between light treatments is not supported. (TIF) [file pone.0029570.s007.tif]

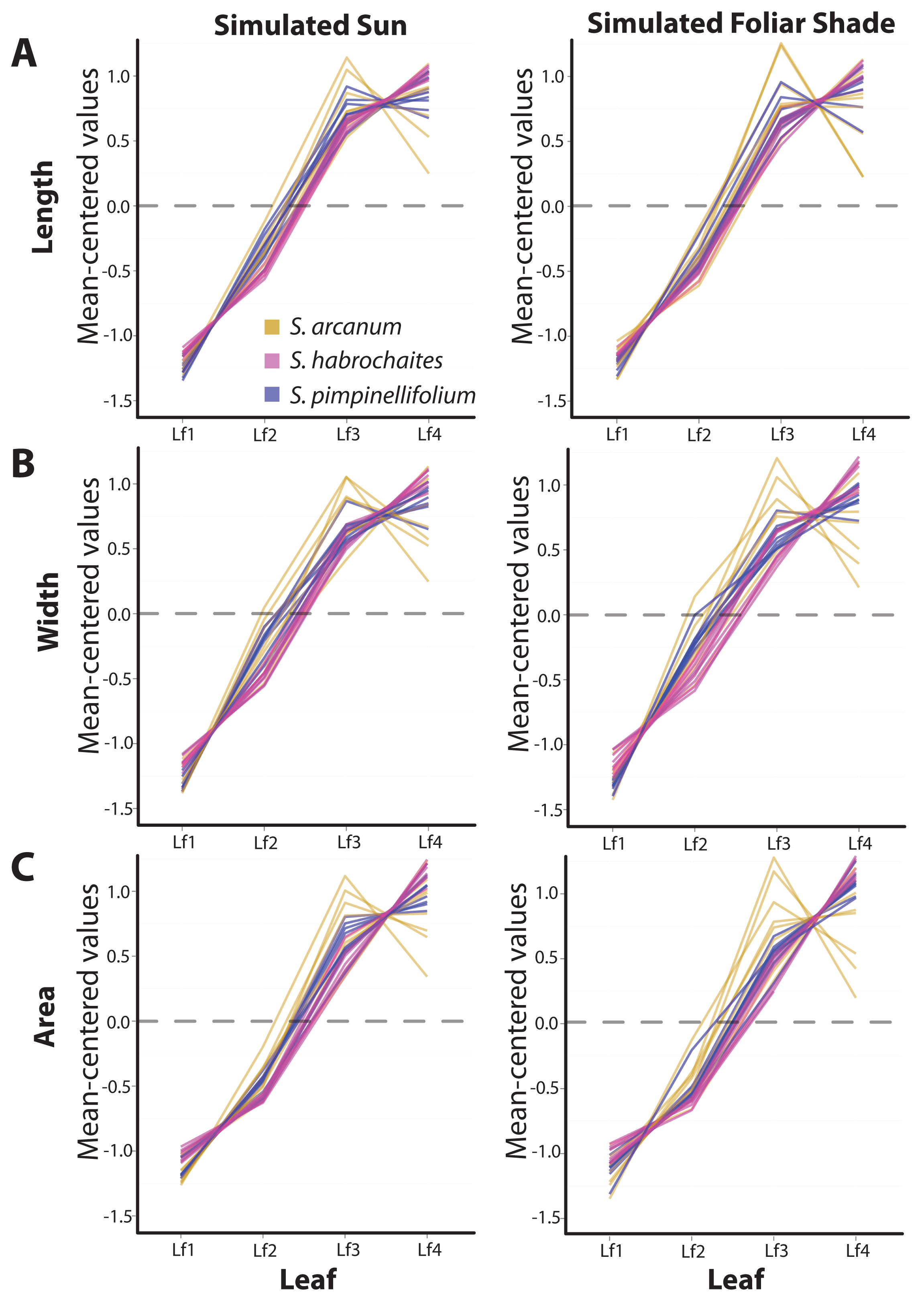

Supplement: Figure S8 — Variation in leaf dimensions across the leaf series amongst accessions. Line graphs for mean leaf A) length, B) width, and C) area values across the leaf series for accessions under simulated sun and shade treatments. Values across the series have been mean-centered at zero, to better reflect changes in the pattern of leaf size across the series rather than overall differences in size. Although there is an overall trend of increasing leaf size through the series, accessions vary as to which leaves in the series are most prominent. Together with the differences in length-to-width ratio observed in leaves at different nodes (Fig. S7), these data may explain the differing correlations in shade avoidance of Sum Length and (Sum Area)1/2 observed with NDVI (as discussed in the text and Fig. 6 ). S. arcanum, S. habrochaites, and S. pimpinellifolium accessions are represented by gold, magenta, and navy, respectively. (TIF) [file pone.0029570.s008.tif]

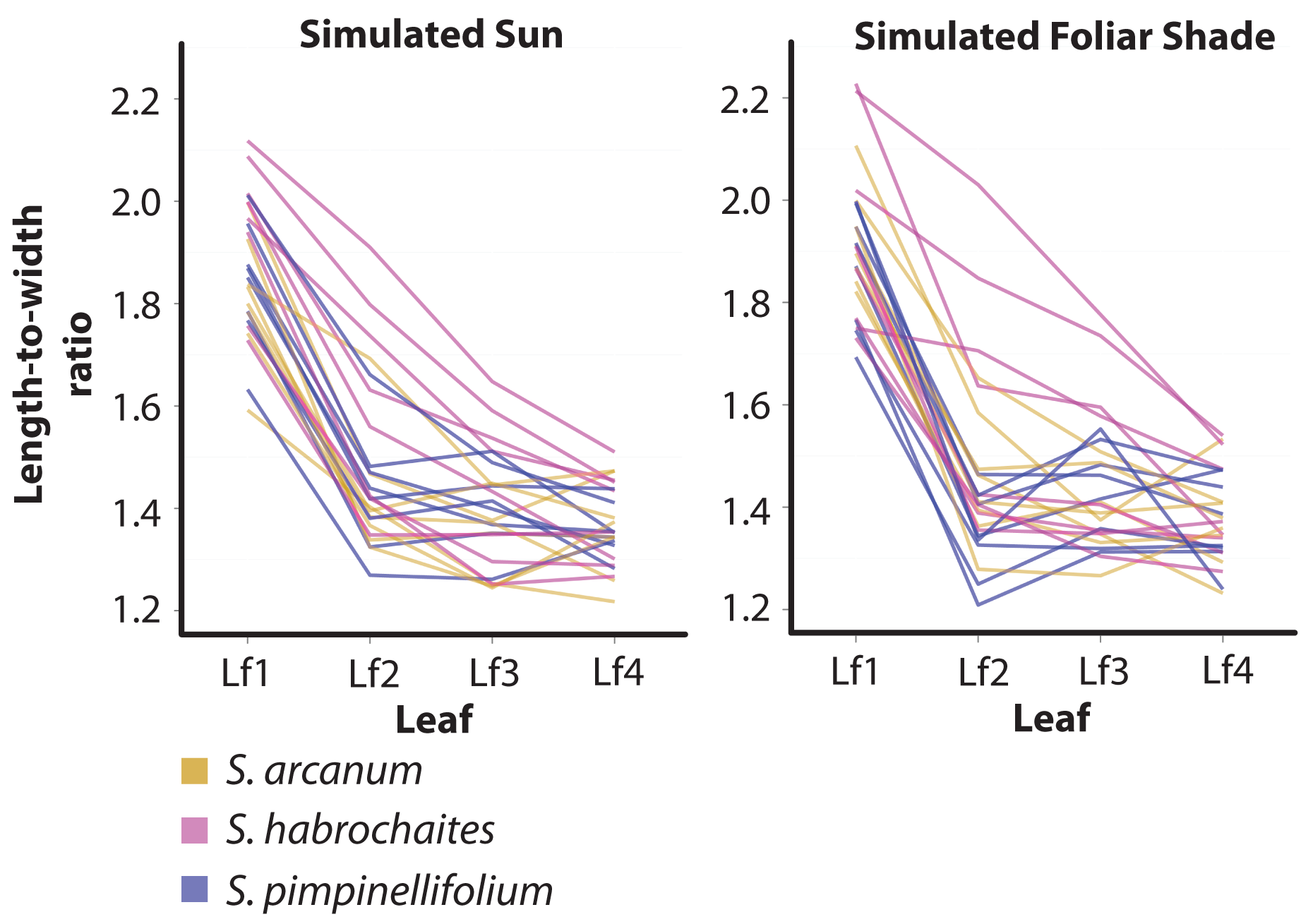

Supplement: Figure S9 — Differences between accessions in the length-to-width ratio of leaves. Mean length-to-width ratio versus leaf node under simulated sun and shade conditions for different accessions. S. arcanum, and S. habrochaites, and S. pimpinellifolium accessions are represented by gold, magenta, and navy, respectively. (TIF) [file pone.0029570.s009.tif]

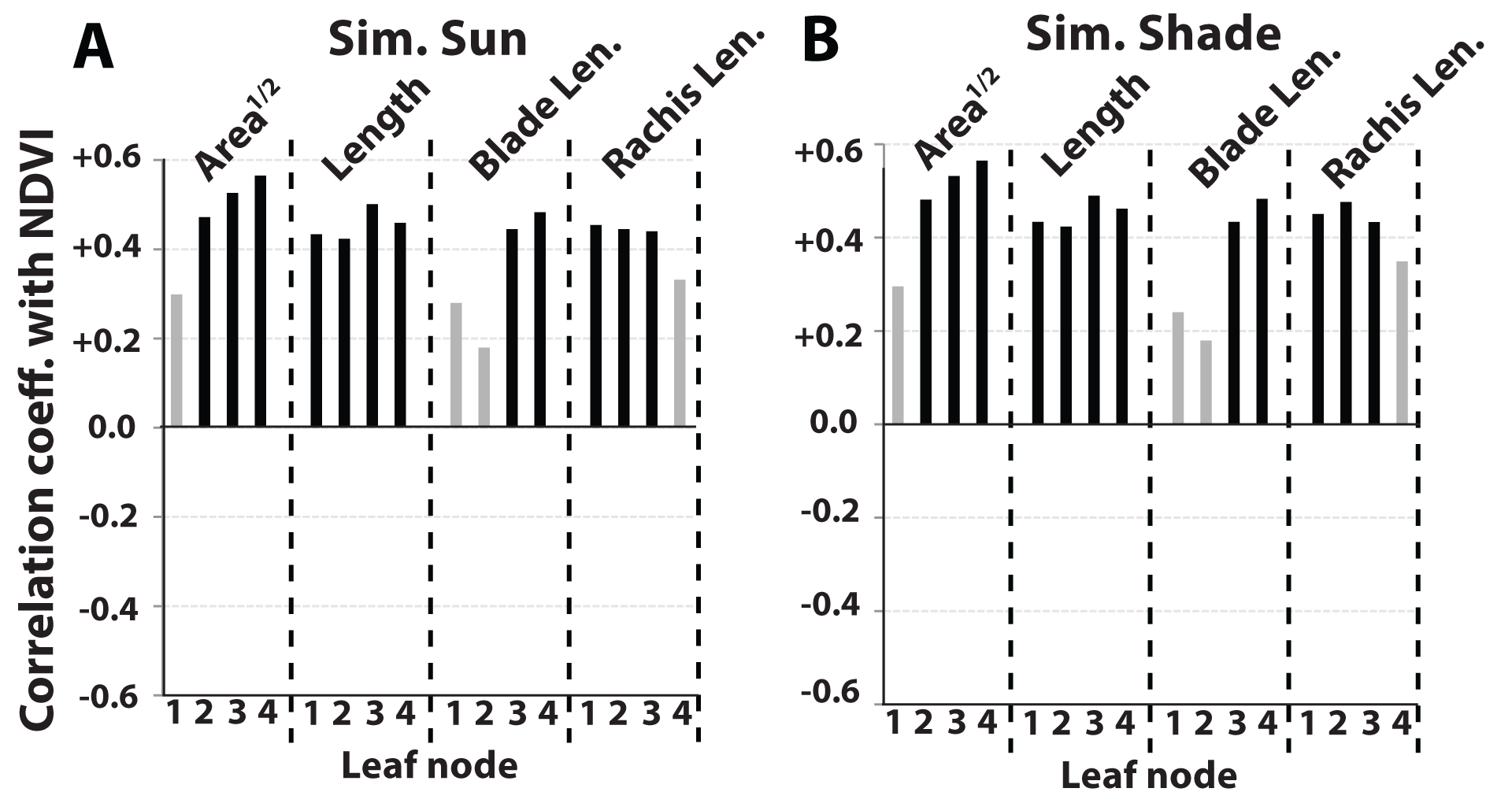

Supplement: Figure S10 — Positive correlation between leaf traits and NDVI across the leaf series. r values representing correlation between leaf dimension traits and sub-regions of the length of the proximal-distal axis with NDVI under A) simulated sun and B) simulated shade conditions. Significance of r values deviating from 0 is denoted by solid (p<0.05) and gray (p>0.05) fill. (TIF) [file pone.0029570.s010.tif]

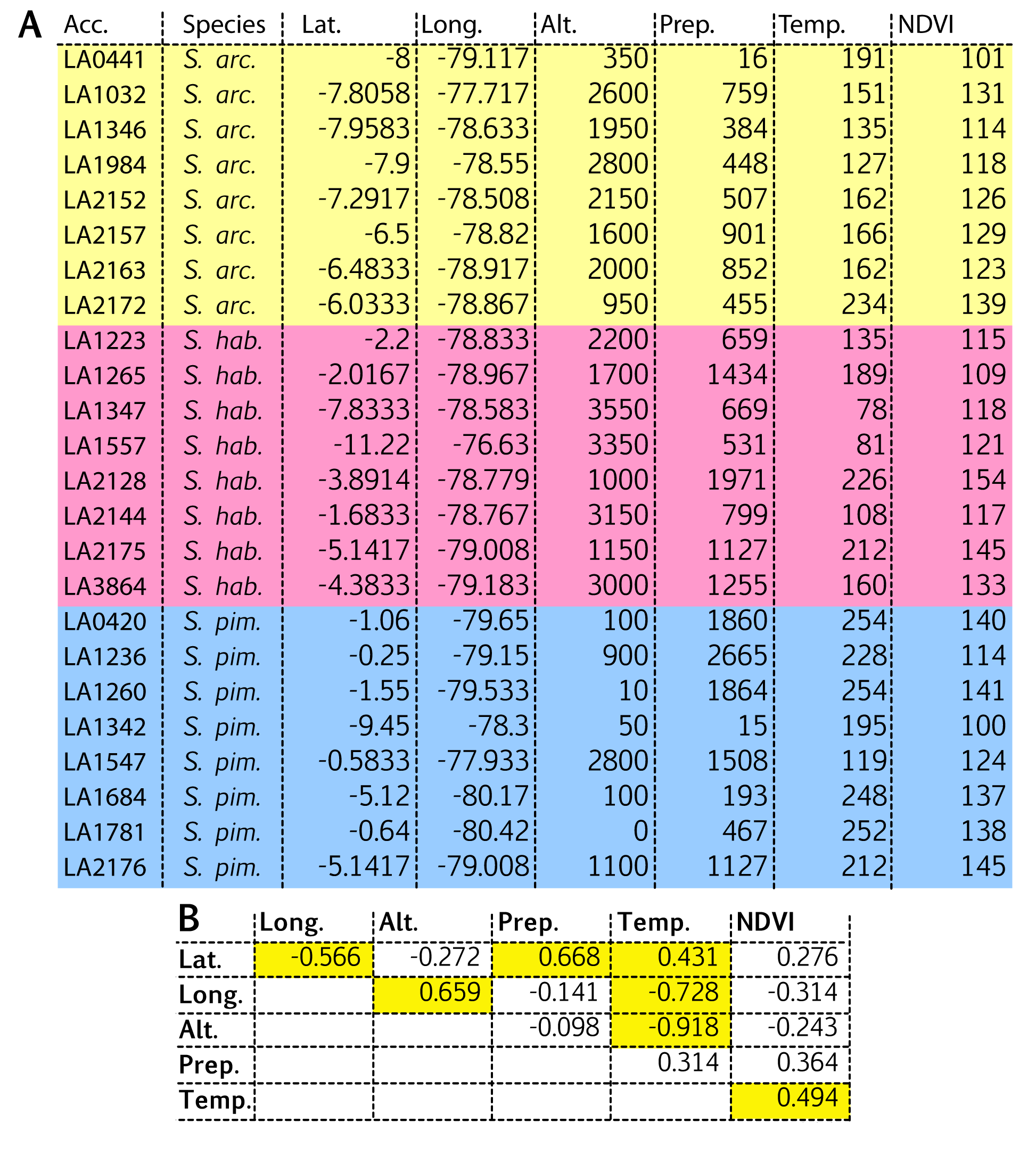

Supplement: Table S2 — Attributes of accessions and inter-correlation. A) Table of latitude and longitude (degrees), altitude (m), mean annual precipitation (mm), mean annual temperature (°C*10), and NDVI values for accessions used in this study. S. arcanum (yellow), S. habrochaites (red), and S. pimpinellifolium (blue) accessions are denoted by their respective colors. B) Table showing correlation coefficients between environmental variables. Significant correlations are highlighted in yellow. (TIF) [file pone.0029570.s012.tif]
