# Supplementary material for: Native Environment Modulates Leaf Size and Response to Simulated Foliar Shade across Wild Tomato Species
Source: PLoS One. 2012 Jan 12;7(1):e29570. doi: 10.1371/journal.pone.0029570 (PMC3257252; doi:10.1371/journal.pone.0029570)
Supplement: Table S1 — p-values for significant terms in the mixed-effect linear models used in this study. Please see Material & Methods in text for details. (PDF) [file pone.0029570.s011.pdf]

Abbreviations:
trt = treatment (simulated sun or simulated foliar shade)
species = species (*S. arcanum*, *S. habrochaites*, or *S. pimpinellifolium*)
acc = accession
leafnos = number of counted leaves
PSQA = perimeter/(area)$^{1/2}$
spe and trt are fixed effects
all other variables are random effects

**Sum length**	p-value
trt	8.56×10^{-5}
species	5.17×10^{-4}
acc	$<2.2 \times 10^{-16}$
ln(leafnos)	$<2.2 \times 10^{-16}$
tray	1.56×10^{-5}

**(Sum area)$^{1/2}$**	p-value
trt	2.80×10^{-4}
species	1.08×10^{-2}
acc	$<2.2 \times 10^{-16}$
ln(leafnos)	$<2.2 \times 10^{-16}$
tray	1.60×10^{-4}

**Sum width**	p-value
trt	2.42×10^{-4}
species	7.74×10^{-3}
acc	$<2.2 \times 10^{-16}$
ln(leafnos)	$<2.2 \times 10^{-16}$
tray	7.91×10^{-7}

**(Leaflet area)$^{1/2}$**	p-value
trt	1.76×10^{-5}
species	3.11×10^{-2}
acc	$<2.2 \times 10^{-16}$
ln(leafnos)	1.91×10^{-3}

**Leaf PSQA**	p-value
trt	1.69×10^{-2}
acc	$<2.2 \times 10^{-16}$
ln(leafnos)	4.32×10^{-9}

**Leaflet PSQA**	p-value
trt	2.49×10^{-2}
species	1.12×10^{-2}
acc	1.32×10^{-2}
ln(leafnos)	3.15×10^{-2}

**Red-to-green ratio**	p-value
trt	4.13×10^{-6}
acc	$<2.2 \times 10^{-16}$
exp	$<2.2 \times 10^{-16}$

**Models for per leaf data:**	p-value	p-value	p-value	p-value
	**Leaf 1 length**	**Leaf 2 length**	**Leaf 3 length**	**Leaf 4 length**
trt	4.27×10^{-5}	8.43×10^{-7}	6.97×10^{-6}	3.57×10^{-5}
species	3.40×10^{-3}	6.22×10^{-5}	1.27×10^{-3}	6.10×10^{-10}
acc	$<2.2 \times 10^{-16}$	$<2.2 \times 10^{-16}$	$<2.2 \times 10^{-16}$	$<2.2 \times 10^{-16}$
ln(leafnos)	8.42×10^{-3}	4.49×10^{-2}	$<2.2 \times 10^{-16}$	$<2.2 \times 10^{-16}$
tray	2.53×10^{-2}	1.44×10^{-2}	3.14×10^{-4}	1.61×10^{-8}

**Models for per leaf data:**	p-value	p-value	p-value	p-value
	**Leaf 1 (area)$^{1/2}$**	**Leaf 2 (area)$^{1/2}$**	**Leaf 3 (area)$^{1/2}$**	**Leaf 4 (area)$^{1/2}$**
trt	3.74×10^{-5}	8.63×10^{-6}	1.12×10^{-4}	1.45×10^{-3}
species	1.82×10^{-4}	3.25×10^{-4}	4.42×10^{-3}	1.29×10^{-2}
acc	$<2.2 \times 10^{-16}$	$<2.2 \times 10^{-16}$	$<2.2 \times 10^{-16}$	$<2.2 \times 10^{-16}$
ln(leafnos)	3.89×10^{-2}	2.80×10^{-2}	4.29×10^{-11}	$<2.2 \times 10^{-16}$
tray	2.44×10^{-2}	1.92×10^{-2}	7.39×10^{-3}	2.47×10^{-4}
	**Leaf 1 rachis length**	**Leaf 2 rachis length**	**Leaf 3 rachis length**	**Leaf 4 rachis length**
trt	6.64×10^{-4}	1.71×10^{-6}	3.19×10^{-5}	5.27×10^{-4}
species	6.91×10^{-3}	1.16×10^{-3}	7.92×10^{-3}	7.65×10^{-10}
acc	$<2.2 \times 10^{-16}$	$<2.2 \times 10^{-16}$	$<2.2 \times 10^{-16}$	1.14×10^{-14}
ln(leafnos)	9.80×10^{-3}	3.81×10^{-3}	$<2.2 \times 10^{-16}$	$<2.2 \times 10^{-16}$
tray	2.55×10^{-2}	3.03×10^{-2}	4.81×10^{-4}	5.32×10^{-9}
	**Leaf 1 terminal blade length**	**Leaf 2 terminal blade length**	**Leaf 3 terminal blade length**	**Leaf 4 terminal blade length**
trt	1.72×10^{-6}	3.48×10^{-7}	1.26×10^{-6}	3.67×10^{-6}
species	8.43×10^{-5}	2.64×10^{-7}	8.24×10^{-5}	3.06×10^{-6}
acc	$<2.2 \times 10^{-16}$	8.07×10^{-14}	$<2.2 \times 10^{-16}$	$<2.2 \times 10^{-16}$
ln(leafnos)	4.56×10^{-2}	1.76×10^{-2}	3.66×10^{-3}	$<2.2 \times 10^{-16}$
tray	2.51×10^{-4}	1.44×10^{-2}	1.04×10^{-3}	7.27×10^{-5}
